# The Application Value of Lipoprotein Particle Numbers in the Diagnosis of HBV-Related Hepatocellular Carcinoma with BCLC Stage 0-A

**DOI:** 10.3390/jpm11111143

**Published:** 2021-11-04

**Authors:** Duo Zuo, Haohua An, Jianhua Li, Jiawei Xiao, Li Ren

**Affiliations:** Department of Clinical Laboratory, Tianjin Medical University Cancer Institute and Hospital, National Clinical Research Center for Cancer, Tianjin’s Clinical Research Center for Cancer, Key Laboratory of Cancer Prevention and Therapy, Tianjin 300060, China; duozuo@tmu.edu.cn (D.Z.); AHH1122@tmu.edu.cn (H.A.); lijianhua@tjmuch.com (J.L.); xiaologin233@163.com (J.X.)

**Keywords:** lipidomics, ^1^H-NMR, LC-MS/MS, lipoprotein subfractions, lipoprotein lipase, cancer biomarkers, hepatocellular carcinoma

## Abstract

Early diagnosis is essential for improving the prognosis and survival of patients with hepatocellular carcinoma (HCC). This study aims to explore the clinical value of lipoprotein subfractions in the diagnosis of hepatitis B virus (HBV)-related HCC. Lipoprotein subfractions were detected by ^1^H-NMR spectroscopy, and the pattern-recognition method and binary logistic regression were performed to classify distinct serum profiles and construct prediction models for HCC diagnosis. Differentially expressed proteins associated with lipid metabolism were detected by LC-MS/MS, and the potential prognostic significance of the mRNA expression was evaluated by Kaplan–Meier *survival analysis*. The diagnostic panel constructed from the serum particle number of very-low-density lipoprotein (VLDL), intermediate-density lipoprotein (IDL), and low-density lipoprotein (LDL-1~LDL-6) achieved higher accuracy for the diagnosis of HBV-related HCC and HBV-related benign liver disease (LD) than that constructed from serum alpha-fetoprotein (AFP) alone in the training set (AUC: 0.850 vs. AUC: 0.831) and validation set (AUC: 0.926 vs. AUC: 0.833). Furthermore, the panel achieved good diagnostic performance in distinguishing AFP-negative HCC from AFP-negative LD (AUC: 0.773). We also found that lipoprotein lipase (LPL) transcript levels showed a significant increase in cancerous tissue and that high expression was significantly positively correlated with the poor prognosis of patients. Our research provides new insight for the development of diagnostic biomarkers for HCC, and abnormal lipid metabolism and LPL-mediated abnormal serum lipoprotein metabolism may be important factors in promoting HCC development.

## 1. Introduction

Hepatocellular carcinoma (HCC) is increasingly recognized as a serious, worldwide public health concern. It is the sixth-most common malignancy and the fourth leading cause of cancer death in the world, with approximately 841,000 new cases and 782,000 deaths worldwide each year. The most common risk factors for HCC are chronic infection with hepatitis B virus (HBV) and hepatitis C virus (HCV); in the vast majority of cases, HCC occurs in individuals with cirrhosis caused by chronic infection with HBV in China [1]. China has the largest liver disease patient population in the world; according to a statistical study of Chinese HCC patients, HBV-related cirrhosis is the most important cause of HCC in China, and more than 80% of HCC patients have varying degrees of HBV infection [2].

Unfortunately, due to a lack of typical clinical manifestations, HCC is difficult to diagnose in the early stage. It has been reported that the overall median survival time of advanced-stage HCC is only 6–10 months; however, for early-stage HCC, surgical treatments such as radiofrequency ablation, selective hepatectomy, and liver transplantation can increase the 5-year survival rate to 60–80% [3]. Therefore, early diagnosis and timely surgical treatment are crucial for better patient outcomes. Pathological examination is the gold standard for diagnosing HCC; however, this method is invasive and is accompanied by risks of bleeding and needle track seeding, which are not recommended before surgery [4]. To date, alpha-fetoprotein (AFP) is the most commonly used serological test for the early diagnosis and monitoring of the development of HCC; however, owing to a lack of sensitivity and specificity, the application of AFP in the diagnosis and prognosis monitoring of HCC is limited [5].

Metabolic reprogramming is recognized as a hallmark in cancer development. Tumor cells must adjust their own metabolic states to maintain excessive proliferation rates; compared with normal cells, the metabolic activities of tumor cells are more vigorous, increasing tumor cell growth and invasion [6]. The associated changes in the metabolite network structure of tumor cells indicate that cancer biomarkers should not be assessed with regard to changes in one or several biochemical indicators but rather to changes in a set of metabolite indicators [7]. The emergence of metabolomics, a discipline that studies the small-molecule intermediates of metabolism in organisms at a certain time [8], has promoted the study of cancer metabolism. As an important branch of metabolomics, lipidomics describes spatial and temporal alterations in the content and composition of different lipid molecules and serves as a powerful tool in the development of lipid biomarkers for studying disease states [9]. Lipidomics has an extremely important position in cancer research; through the high-throughput detection and quantitative analysis of biological fluids (blood, urine, saliva, and fecal extracts), lipidomics can be used to study the mechanism of disease occurrence and development [10].

^1^H-nuclear magnetic resonance (^1^H-NMR) is one of the most commonly used high-throughput platforms in metabolomics research. ^1^H-NMR spectroscopy provides an alternative method of measuring lipoprotein levels in serum, and quantitative detection by ^1^H-NMR can determine the quality, particle number, and particle size of lipoprotein subfractions by detecting the terminal methyl protons of phospholipids, unesterified cholesterols, cholesterol esters and triglycerides [11]. As potential risk factors for HCC development, hepatitis and liver cirrhosis are often associated with serum lipid and lipoprotein aberrations, and a number of reports have illustrated that the serum levels of many kinds of lipids, lipoproteins and apolipoproteins show obvious changes in HCC patients [12,13]. Lipoprotein particle distributions have great potential for helping improve the diagnostics of metabolic disorders [14]; however, studies estimating subfractions of lipoproteins have been restricted to patients with cardiovascular disease and are rarely extended to the exploration of cancer research [15,16,17]. Previous serum and urine metabolomics studies have illustrated that compared with those of patients with cirrhosis or healthy controls, several small-molecule metabolites, such as glucose, glutamine, citrate, creatine, creatinine, carnitine, glycine, and acetate, show remarkable changes in HCC patients [18,19,20,21]. However, few studies have focused on lipid metabolism disturbances and serum lipoprotein subfraction changes in HBV-related HCC patients. Therefore, this study aims to develop novel diagnostic lipid biomarkers of HBV-related HCC.

In this article, we utilized ^1^H-NMR to detect collected serum samples and performed multivariable and univariable statistical analysis to study the serum lipoprotein subfraction in patients with HBV-related HCC, patients with benign liver disease (including HBV-related hepatitis and HBV-related cirrhosis) and healthy patients. The aim of our study was to identify serum lipidome-based biomarkers as a diagnostic multivariable model for early-stage HCC. Furthermore, we obtained paired cancerous tissues and matched paracancerous tissues from HCC patients to search for differentially expressed proteins involved in lipid metabolism and explore the association with prognosis in patients with HBV-related HCC.

## 2. Results

### 2.1. Clinical Characteristics

The demographic and clinical characteristics of the study participants with HCC and LD are summarized in Table 1. In the training set, the serum levels of AFP, alanine transaminase (ALT), aspartate transaminase (AST), and total protein (TP) were significantly different between the HCC and LD groups (*p* < 0.05). In the validation set, compared with those of patients with LD, the serum AFP levels of the HCC patients were significantly different (*p* < 0.05). The clinical characteristics of the normal controls (NCs) and AFP-negative patients are summarized in Tables S2 and S3.

### 2.2. NMR Spectroscopic Multivariable Analysis

For comprehensive observation of the lipoprotein subfractions, PCA and PLS-DA were employed to explore the intrinsic differences between different groups. The score plot of PCA and PLS-DA (Figure S2) showed that lipoprotein subfractions of the three groups could be distinguished, and the model parameters of PCA (R^2^X = 0.99, Q^2^ = 0.944) and PLS-DA (R^2^Y = 0.447, Q^2^Y = 0.402, CV-ANOVA *p* < 0.0001) indicated that the constructed models have favorable robustness.

**Figure 1:**
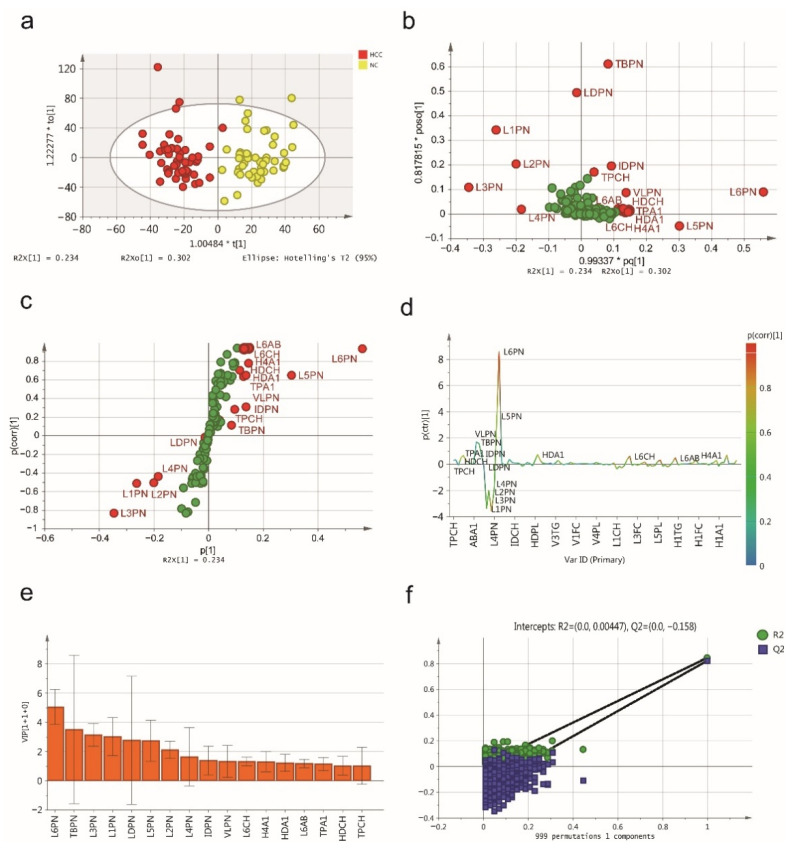
The serum lipidomic profile discriminates between HCC patients and normal controls (NCs). (**a**) Score plot was generated by the OPLS-DA model. The horizontal coordinate (1.00484 * t [1]) represents the score value of the main component, and the vertical coordinate (1.22277 * to [1]) represents the score value of the orthogonal component. (**b**) Loading plot was generated by the OPLS-DA model. The horizontal coordinate (0.99337 * pq [1]) represents the predicted principal component, and the vertical coordinate (0.817815 * poso [1]) represents the orthogonal principal components. The corresponding (**c**) S-plot and (**d**) S-line plot for the model displaying the discriminant variables and the associated predictive loadings. The red circles indicate selected lipoprotein subfractions with VIP scores >1.0, and other variables with no difference are referred to as green circles in (**b**,**c**). (**e**) The selected lipoprotein subfractions with VIP scores >1.0. (**f**) Permutation test (999 times) of the OPLS-DA model.

To further filter the variables, the ^1^H-NMR serum spectra of the patients with HCC and NCs were discriminated with the OPLS-DA model, as shown in a score plot (Figure 1a), which illustrates that this model can significantly discriminate between HCC patients and NCs. The predictive ability was calculated through 7-fold cross-validation (R^2^Y = 0.843, Q^2^Y = 0.821, CV-ANOVA *p* < 0.0001), suggesting that the model possessed a satisfactory fit with good predictive power. The loading plot indicated a brief overview of the contribution of each lipoprotein subfraction to the OPLS-DA model (Figure 1b), and the variables responsible for significantly contributing to the separation of the two groups are indicated in the corresponding S-plot (Figure 1c) and S-line plot (Figure 1d). Using the variable importance in projection (VIP) score (>1.0) from the OPLS-DA model, a total of 17 lipoprotein subfractions were selected (Figure 1e). To further assess the robustness of the constructed OPLS-DA model and prevent it from overfitting, a 999-permutation test (Figure 1f) was performed, and the results (intercepts: R^2^ = 0.138, Q^2^ = −0.313) indicated that this OPLS-DA model had high discriminability.

**Figure 2:**
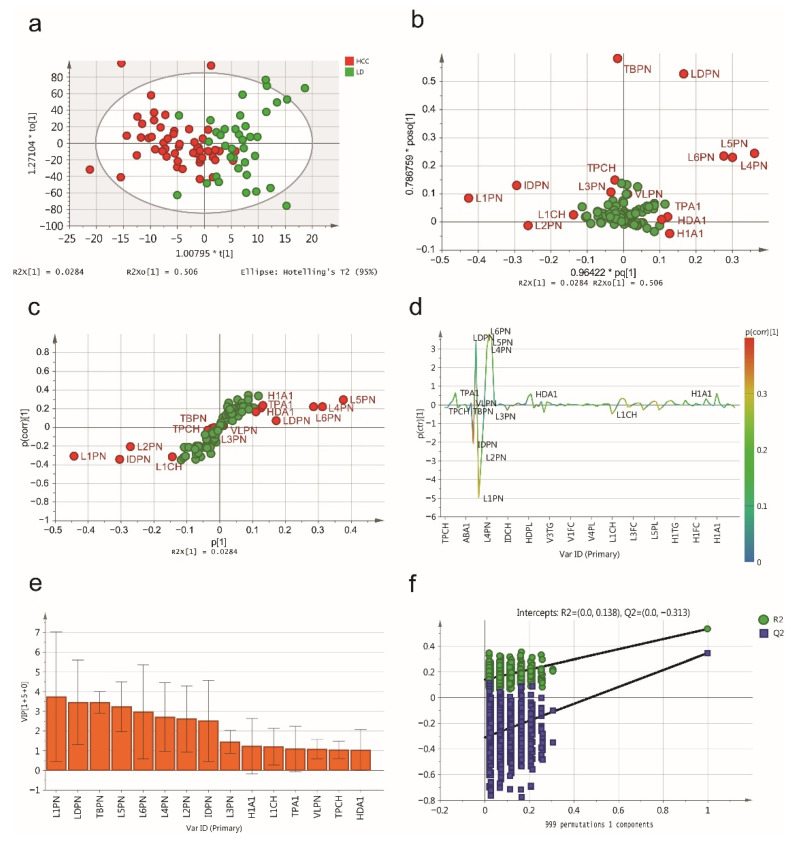
The serum lipidomic profile discriminates between HCC patients and liver disease (LD). (**a**) Score plot was generated by the OPLS-DA model. The horizontal coordinate (1.00795 * t [1]) represents the score value of the main component, and the vertical coordinate (1.27104 * to [1]) represents the score value of the orthogonal component. (**b**) Loading plot was generated by the OPLS-DA model. The horizontal coordinate (0.96422 * pq [1]) represents the predicted principal component, and the vertical coordinate (0.786795 * poso [1]) represents the orthogonal principal components. The corresponding (**c**) S-plot and (**d**) S-line plot for the model displaying the discriminant variables and the associated predictive loadings. The red circles indicate selected lipoprotein subfractions with VIP scores >1.0, and other variables with no difference are referred to as green circles in (**b**,**c**). (**e**) The selected lipoprotein subfractions with VIP scores >1.0. (**f**) Permutation test (999 times) of the OPLS-DA model.

Then, we applied another OPLS-DA model to distinguish HCC patients from LD patients, and the score plot indicated that the group of HCC patients could be excellently separated from the LD patient group (Figure 2a). A loading plot (Figure 2b) illustrated the contribution of each lipoprotein subfraction in distinguishing HCC patients from LD patients, and the S-plot (Figure 2c) and S-line plot (Figure 2d) showed the variables significantly contributing to the separation. According to the VIP score (>1.0), a total of 15 lipoprotein subfractions were selected (Figure 2e). The results of the internal validation (R^2^Y = 0.530, Q^2^Y = 0.343, CV-ANOVA *p* < 0.001) and the results of the permutation test (intercepts: R^2^ = 0.004, Q^2^ = −0.158) suggested that the constructed OPLS-DA model has favorable robustness and could be used in the next step of analysis (Figure 2f). The relevant lipoprotein subfractions and their statistical details are listed in Table 2.

**Figure 3:**
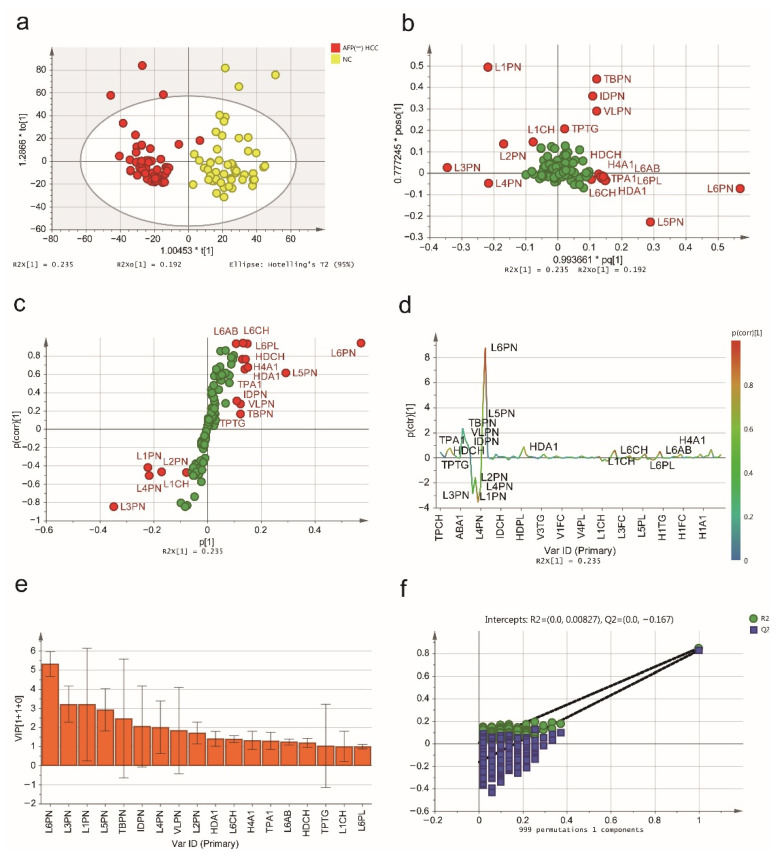
The serum lipidomic profile discriminates between AFP-negative HCC patients and NCs. (**a**) Score plot was generated by the OPLS-DA model. The horizontal coordinate (1.00453 * t [1]) represents the score value of the main component, and the vertical coordinate (1.2866 * to [1]) represents the score value of the orthogonal component. (**b**) Loading plot was generated by the OPLS-DA model. The horizontal coordinate (0.993661 * pq [1]) represents the predicted principal component, and the vertical coordinate (0.777245 * poso [1]) represents the orthogonal principal components. The corresponding (**c**) S-plot and (**d**) S-line plot for the model displaying the discriminant variables and the associated predictive loadings. The red circles indicate selected lipoprotein subfractions with VIP scores >1.0, and other variables with no difference are referred to as green circles in (**b**,**c**). (**e**) The selected lipoprotein subfractions with VIP scores >1.0. (**f**) Permutation test (999 times) of the OPLS-DA model.

Next, the PCA and PLS-DA analysis of the serum AFP-negative patients showed that lipoprotein subfractions of the three groups could be distinguished (Figure S3), with parameters of PCA (R^2^X = 0.992, Q^2^ = 0.947) and PLS-DA (R^2^Y = 0.430, Q^2^Y = 0.390, CV-ANOVA *p* < 0.0001). The ^1^H-NMR serum spectra of the patients with AFP-negative HCC and NCs were discriminated with the OPLS-DA model, as shown in a score plot (Figure 3a), and the predictive ability was calculated through 7-fold cross-validation (R^2^Y = 0.848, Q^2^Y = 0.827, CV-ANOVA *p* < 0.0001). The loading plot (Figure 3b) illustrated the contribution of each lipoprotein subfraction in distinguishing AFP-negative HCC patients from NCs, and the S-plot (Figure 3c) and S-line plot (Figure 3d) showed the variables significantly contributing to the separation. Using the VIP score (>1.0) from the OPLS-DA model, a total of 18 lipoprotein subfractions were selected (Figure 3e). Meanwhile, the results of the permutation test (intercepts: R^2^ = 0.00827, Q^2^ = −0.167, Figure 3f) suggested that the constructed OPLS-DA models have favorable robustness.

**Figure 4:**
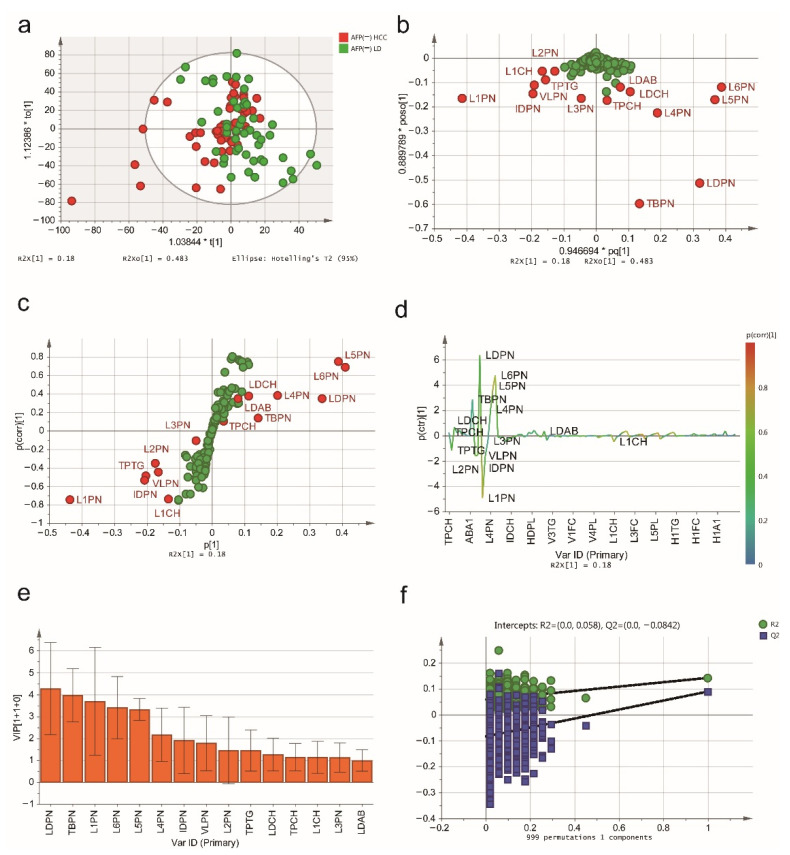
The serum lipidomic profile discriminates between AFP-negative HCC patients and AFP-negative LD patients. (**a**) Score plot was generated by the OPLS-DA model. The horizontal coordinate (1.03844 * t [1]) represents the score value of the main component, and the vertical coordinate (1.12386 * to [1]) represents the score value of the orthogonal component. (**b**) Loading plot was generated by the OPLS-DA model. The horizontal coordinate (0.946694 * pq [1]) represents the predicted principal component, and the vertical coordinate (0.889789 * poso [1]) represents the orthogonal principal components. The corresponding (**c**) S-plot and (**d**) S-line plot for the model displaying the discriminant variables and the associated predictive loadings. The red circles indicate selected lipoprotein subfractions with VIP scores >1.0, and other variables with no difference are referred to as green circles in (**b**,**c**). (**e**) The selected lipoprotein subfractions with VIP scores >1.0. (**f**) Permutation test (999 times) of the OPLS-DA model.

Next, we applied another OPLS-DA model to distinguish AFP-negative HCC patients from AFP-negative LD patients (Figure 4a). The loading plot (Figure 4b) illustrated the contribution of each lipoprotein subfraction in distinguishing these two groups, and the S-plot (Figure 4c) and S-line plot (Figure 4d) showed the variables significantly contributing to the separation. According to the VIP score (>1.0) and S-line plot (Figure 4e) of the OPLS-DA model, a total of 15 lipoprotein subfractions were selected. The results of the internal validation (R^2^Y = 0.142, Q^2^Y = 0.0893, CV-ANOVA *p* = 0.057) and the results of the permutation test (intercepts: R^2^ = 0.058, Q^2^ = −0.0842) suggested that the constructed OPLS-DA model has favorable robustness and could be used in the next step of analysis (Figure 4f). The relevant lipoprotein subfractions and their statistical details are listed in Table 3.

**Figure 5:**
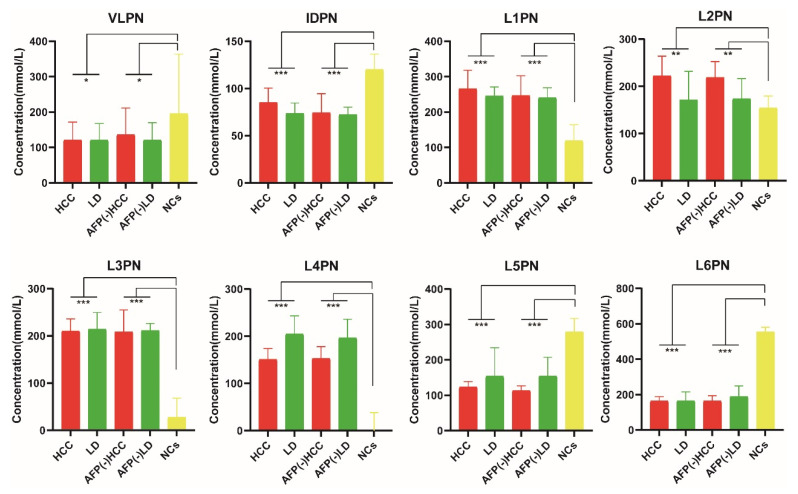
Comparisons between the groups of diagnostic biomarkers. Histograms indicate the median, upper, and lower quartiles of the eight lipoprotein particle numbers used to construct the diagnostic panel. The significance of the values was assessed using the Kruskal–Wallis test (* *p* < 0.05, ** *p* < 0.001, *** *p* < 0.0001). HCC, all HCC patients in the training set; LD, all liver disease patients in the training set; AFP (-) HCC, serum AFP-negative expression HCC patients; AFP (-) LD, serum AFP-negative expression liver disease patients; NCs, normal controls.

To further determine which lipoprotein subfractions could be used as biomarkers for HCC diagnosis, the common variables in these OPLS-DA models (VIP scores > 1.0 and *p*-values < 0.05) were selected for the subsequent analysis, including VLPN, IDPN, and L1-L6PN (Table 4 and Figure 5). Detailed information on all lipoprotein subfractions is listed in Tables S4 and S5. The absolute numbers of each lipoprotein subfraction are listed in Table S6.

### 2.3. Biomarker Selection and Validation of the Diagnostic Model

**Figure 6:**
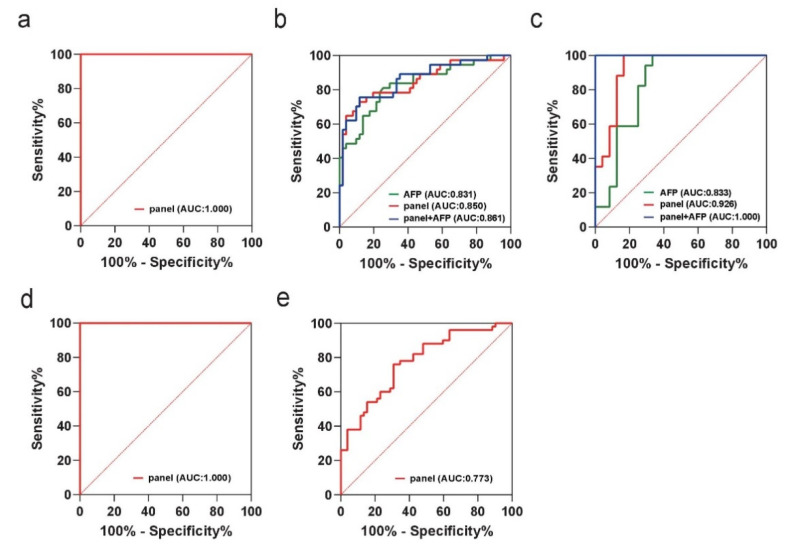
ROC curve analysis following binary logistic regression. The diagnostic performance of the lipidomic biomarker panel in discriminating HCC from (**a**) normal controls (NCs) in the training set. Comparison of the diagnostic performance of the lipidomic biomarker panel with that of serum alpha-fetoprotein (AFP) and their combination in discriminating HCC from liver disease (LD) in the (**b**) training set and (**c**) validation set. The ROC curve of the lipidomic biomarker panel also showed excellent discriminability in discriminating AFP-negative HCC patients from (**d**) NCs and (**e**) AFP-negative LD patients. The red-, green-, and blue-colored lines indicate lipid biomarkers, AFP, and lipid biomarkers with AFP, respectively.

To judge the diagnostic performance of selected variables, binary logistic regression analysis was employed to construct the best diagnostic model. Meanwhile, the correlation analysis showed that serum AFP levels had no significant correlation with the selected variables, indicating that lipoprotein particles and AFP are independent of each other (Figure S4). Meanwhile, we analyzed the correlation of selected variables with clinical features by the nonparametric Spearman correlation test. According to the results, L1PN, L2PN, and L3PN were strongly positively associated with age (the range of Spearman’s rank correlation coefficient was from 0.3 to 0.5, *p*-value < 0.05) and negatively associated with male gender (the range of Spearman’s rank correlation coefficient was from −0.3 to −0.5, *p*-value < 0.05). Furthermore, VLPN was strongly positively associated with tumor size (Spearman’s coefficient r = 0.326, *p* = 0.004) and negatively associated with liver cirrhosis (Spearman’s coefficient r = −0.372, *p* = 0.001). IDPN was strongly positively associated with tumor size (Spearman’s coefficient r = 0.306, *p* = 0.008). The results are shown in Figure S5 and Table S7. According to the results of ROC curve analysis, the panel composed of VLPN, IDPN, and L1-L6PN reached excellent diagnostic performance in discriminating HCC patients from NCs with an AUC of 1.000 (95% CI: 0.964–1.000) (Figure 6a). Furthermore, the panel showed better diagnostic performance than serum AFP alone in discriminating HCC patients from LD patients, as indicated by an AUC of 0.850 (95% CI: 0.758–0.917) vs. 0.831 (95% CI: 0.736–0.902), respectively, in the training set, and combining the lipidomic biomarkers with AFP increased the AUC to 0.861 (95% CI: 0.771–0.926) (Figure 6b).

The diagnostic performance of the panel was further confirmed in the external validation set. The diagnostic accuracy of this panel in the validation cohort also demonstrated a superior performance to serum AFP alone (AUC: 0.926; 95% CI: 0.800–0.984 vs. AUC: 0.833; 95% CI: 0.684–0.931), and their combination increased the AUC to 1.000 (95% CI: 0.914–1.000) (Figure 6c). Meanwhile, this panel also achieved good diagnostic accuracy in discriminating AFP-negative HCC patients from NCs and AFP-negative LD patients, with AUCs of 1.000 (0.964–1.000) (Figure 6d) and 0.773 (0.680–0.850) (Figure 6e), respectively. The ROC results are shown in Table 5. Furthermore, to investigate whether the diagnostic panel can more realistically reflect the diagnostic approach in regular patient care, we unified LD patients and NCs into the non-HCC group (*n* = 104) and performed a differential diagnosis analysis of the non-HCC group versus the HCC group (*n* = 75). According to binary logistic regression and ROC curve analysis, the panel constructed by these eight indicators achieved good diagnostic accuracy (AUC: 0.842; 95% CI: 0.780–0.892). Meanwhile, we unified AFP-negative LD patients and NCs into the AFP (-) non-HCC group (*n* = 100) and compared them with AFP-negative HCC patients (*n* = 52). The panel constructed by these eight indicators also achieved good diagnostic accuracy (AUC: 0.837; 95% CI: 0.769–0.892) in determining serum AFP-negative expression populations. The ROC results are shown in Table S8 and Figure S6.

These results indicate that the panel constructed by VLPN, IDPN, and L1-L6PN has strong potential in the diagnosis of HBV-related HCC. ^1^H-NMR-based quantitative analysis of serum lipoprotein subfractions thus has potential in clinical applications for discovering specific novel diagnostic biomarkers of HBV-related HCC.

### 2.4. Lipoprotein Lipase (LPL) Is Upregulated in HCC and Associated with Poor Prognosis

**Figure 7:**
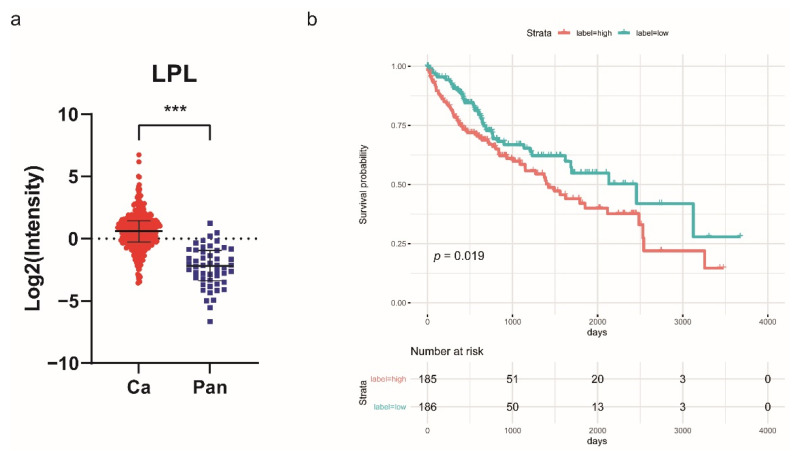
LPL levels are upregulated in HCC tissues and associated with poor prognosis. (**a**) LPL mRNA levels in HCC (red circle) and adjacent normal (blue square) tissues in the TCGA-LIHC dataset. The *p*-value was assessed using the Mann–Whitney U test (*** *p* < 0.0001). (**b**) Overall survival analysis was plotted using the TCGA database at the threshold *p*-value of < 0.05.

VLDL is hydrolyzed by LPL to generate smaller denser particles and subsequently IDL in the peripheral circulation, which is converted to LDL by further hydrolysis. Our results showed that the serum VLDL and IDL levels of HCC patients decreased significantly, while the serum LDL1, LDL2, LDL3, and LDL4 levels of HCC patients increased significantly. The reason for this phenomenon may be related to the increased secretion of LPL into the peripheral blood by tumor cells in HCC patients. We analyzed the LPL transcript level data of HCC patients in the TCGA database and found that LPL mRNA expression level in cancerous tissues of HCC patients showed a significant increase compared with paracancerous tissues (Figure 7a), and the high expression of LPL showed a significant positive correlation with the poor prognosis of patients (Figure 7b), suggesting that abnormal lipoprotein metabolism due to upregulation of LPL mRNA expression in liver tissues may be related to the development of HCC.

### 2.5. Identification of Differentially Expressed Protein and Lipid Metabolism-Related Pathways

Through LC-MS/MS platform analysis, a total of 5393 proteins were identified in eight cancerous and paracancerous tissue samples. By employing an FDR adjusted *p*-value of 0.01, a fold-change value >1.5 or <0.5 and a *p*-value < 0.05 as cutoff values, a total of 11 differentially expressed proteins (DEPs) associated with lipid metabolism were detected. Among these, four proteins were significantly upregulated in HCC tissue, including ACSL4, MBOA7, ACLY, and GPDM. In contrast, seven proteins were downregulated, including GPDA, ACOX2, ECHM, ACADS, CP2C9, H17B6, and CP39A (Figures S7 and S8). Through further pathway enrichment analysis, we found that the genes that regulate these differential proteins were highly enriched in fatty acid biosynthesis, glycerophospholipid metabolism, primary bile acid biosynthesis, arachidonic acid metabolism and steroid hormone biosynthesis (Figure S9 and Table S9). Moreover, we evaluated the potential prognostic significance of the mRNA expression of the genes encoding these proteins using data from the GEPIA database (http://gepia2.cancer-pku.cn/ accessed on 23 August 2021). Kaplan–Meier survival analysis revealed that high expression of MBOAT7 and GPD2 and low expression of ACADS were associated with a poor prognosis (Figure S10).

## 3. Discussion

A definite differential diagnosis between early-stage HBV-related HCC and HBV-related benign LDs, such as HBV-related hepatitis and HBV-related cirrhosis, is often difficult due to a lack of obvious clinical, serological, or radiological evidence. At present, AFP remains a widely used tumor-specific serological biomarker in the diagnosis and management of HCC. However, high AFP expression may be detected in certain pathological conditions, such as deterioration of chronic liver disease, pregnancy, and the presence of germ cell tumors or gastric cancer [5].

^1^H-NMR spectroscopy is the most commonly used detection platform in the application of lipidomics; despite its lower sensitivity, NMR spectroscopy has several unique advantages over mass spectrometry (MS). ^1^H-NMR is a noninvasive testing technology that has excellent cross-laboratory reproducibility and does not require elaborate sample preparation or fractionation [8,22]. Routine lipid detections (such as the tests of serum levels of total cholesterol (TC), triglycerides (TG), LDL cholesterol (LDL-C), and HDL cholesterol (HDL-C)) are conventionally used in the clinical analysis of circulating lipid metabolites. ^1^H-NMR spectroscopy is a rapid, alternative method for quantifying lipoproteins; through the detection of amplitudes of spectral signals emitted by lipoprotein subfractions, one can obtain a direct indication of subclass particle concentration [11]. In this study, we utilized a ^1^H-NMR high-throughput platform to detect serum lipoprotein subfractions in HBV-related HCC patients with BCLC stage 0-A, at-risk populations (HBV-related hepatitis and cirrhosis) and a healthy control population. In the selection of biomarkers and validation of the diagnosis model, OPLS-DA showed a distinct separation of HCC patients from benign LD patients and NCs. Furthermore, the OPLS-DA model achieved good accuracy for HCC patients relative to normal controls. However, in the OPLS-DA model constructed by the HCC and LD subjects, the Q^2^ value failed to reach the desired cutoff level, which might be because of the fewer differences in the metabolic patterns due to the similarity of liver function status between early-stage HCC and LD patients. After multivariable and univariable statistical analyses, a total of eight lipoprotein particle numbers, including VLPN, IDPN, L1PN, L2PN, L3PN, L4PN, L5PN, and L6PN, were selected to build the diagnostic panel. Compared with the serum AFP level alone, the panel constructed from the different lipoprotein particle numbers achieved a higher accuracy in discriminating HCC in the training set and validation set than AFP alone. We also found that the panel achieved excellent diagnostic performance in discriminating AFP-negative HCC patients from AFP-negative LD patients and NCs.

The liver is the major organ of energy metabolism and plays a central role in lipoprotein metabolism by regulating the balance between β-oxidation and lipid synthesis [23]. Most serum endogenous lipids and lipoproteins are synthesized in the liver. The main function of lipoproteins is to transport lipids between cells, which are critical in maintaining energy homeostasis as well as the pathogenesis of atherosclerosis [24]. Under normal physiological conditions, the liver ensures homeostasis of lipid and lipoprotein metabolism, which depends on the structural and functional integrity of hepatocytes [25,26]. However, due to their increased demand for lipids, tumor cells show increased extracellular lipid uptake and a high de novo lipid synthesis rate, which is necessary for HCC tumorigenesis, survival, and progression [27]. De novo lipogenesis starts with the conversion of citrate to oxaloacetate and acetyl-coenzyme A (CoA), which is mediated by ATP-citrate lyase (encoded by ACLY) [28]. Acetyl-CoA is converted to malonyl-CoA via acetyl-CoA carboxylase (ACC) and then to saturated fatty acids (FAs) through the action of fatty acid synthase (encoded by FASN) [28]. HCC is typically characterized by the aberrant overexpression of enzymes in this process, such as ACLY, ACC, and FASN [28,29]. The mass spectrometry results showed that ACLY and MBOA7 were significantly elevated in HCC patients’ cancerous tissues, indicating that the upregulation of de novo lipid synthesis was associated with HCC tumorigenesis [30]. In particular, we noted that the level of long-chain ACSL4 expression in HCC cancerous tissue was significantly higher than that in paracancerous tissue (fold change = 15.59), and members of the ACSL family are key enzymes involved in the initial steps of FA metabolism, converting FA to fatty acyl-CoA esters [31]. As a member of the ACSL family, ACSL4 is poorly expressed in the organs of the gastrointestinal system, such as the liver. Chen et al. found that ACSL4 is frequently upregulated in HCC tissues compared with normal samples and promotes HCC progression via c-Myc stability mediated by the ERK/FBW7/c-Myc axis [32].

Under physiological conditions, lipid components such as triglycerides and cholesterol are transported as lipoproteins in the peripheral blood. Among them, exogenous lipids are absorbed through the intestinal epithelium and synthesized as celiac particles (CMs), endogenous lipids entering the liver and synthesized as VLDL, both collectively known as triglyceride-rich lipoproteins (TRLs) [33,34]. The newly secreted TRL enters the bloodstream and needs to be marginalized along the luminal surface of capillaries and hydrolyzed by lipoprotein lipase (LPL) expressed on the surface of vascular endothelial cells from TG within the neutral core of CMs and VLDL to produce CMs residue and IDL, respectively, and release free fatty acids (FFA) for use by peripheral tissues, where IDL can be absorbed by the liver or through further TG hydrolysis to LDL [35,36]. Adipocytes, cardiomyocytes, and skeletal muscle cells are the main sites for producing LPL. Because these cells are far away from the capillary cavity and need to be transported through the subendothelial space, recent studies have shown that glycosylphosphatidylinositol anchors high-density lipoprotein binding protein 1 (GPHIBP1), which captures LPL and binds to form the LPL–GPDIBP1 complex to mediate LPL entry into the lumen through capillary endothelial cells and specifically binds to ApoCII in TRL to exert a hydrolytic effect [36,37]. Recent studies have shown that LPL expression appears upregulated in several types of tumor cells and is associated with cancer progression and poor prognosis. In our study, the serum VLDL and IDL levels of HCC patients decreased significantly, while the serum LDL1, LDL2, LDL3, and LDL4 levels of HCC patients increased significantly. We speculated that this phenomenon may be related to the increased secretion of LPL into the peripheral blood by tumor cells in HCC patients. Therefore, we analyzed and found that the LPL mRNA expression level in cancerous tissues of HCC patients showed a significant increase compared with paracancerous tissues, and the high expression of LPL showed a significant positive correlation with the poor prognosis of HCC patients from TCGA database. Cao et al. found that the mRNA and protein expression levels of LPL were upregulated in mouse and human HCC tissues and positively correlated with poor prognosis, and in vitro experiments further showed that culturing cells in the absence or silencing of LPL significantly reduced cell proliferation [38]. This is consistent with our findings. Wu et al. found that the expression of the antioncogene ZHX2 was significantly reduced in nonalcoholic fatty liver disease (NAFLD)-associated HCC and that overexpression of ZHX2 inhibited the uptake of exogenous lipids and the ability of HCC cells to proliferate by suppressing LPL promoter activity, thereby delaying the progression of NAFLD-associated HCC [39]. Manupati et al. found that LPL transcript levels were upregulated 16-fold in CD44-positive breast cancer stem cells. LPL, as a unique downstream target of CD44 signal transduction, can activate endothelial cell-mediated angiogenesis during tumor growth. In addition, knockdown of CD44 or intratumoral injection of tetrahydrolipostatin (LPL inhibitor) can inhibit breast cancer progression and angiogenesis [40]. LPL also plays an important role in the production of IDL and LDL in the human body. This suggests that the significant differences in some indicators between the HCC group and the control group may be related to LPL expression, such as VLPN, IDPN, L1PN, L2PN, L4PN, and L5PN. We will explore the correlation in the future study.

The diagnostic panel constructed from serum lipoprotein particle numbers effectively improved the detection of patients with early-stage HCC, illustrating that ^1^H-NMR lipoprotein subfraction testing plays an important role in the diagnosis of early-stage HCC. Lu et al. observed that the L1 and L5 subfractions of LDL and VLDL promoted breast cancer cell migration and invasion through increased Akt Ser473 phosphorylation [41]. Further angiogenic assays in vitro indicated that the L1 and L5 subfractions and VLDL enhanced the secretion of angiogenic factors and promoted angiogenic activity [41]. There are few studies on the mechanisms of lipoprotein subfractions in the tumorigenesis and development of hepatocarcinoma, and we will explore this aspect in the future.

In addition, we recognize some limitations in our research. First, the sample size of the external validation set was relatively small, and the patients were not equally distributed between the HCC and LD groups. Therefore, a sufficiently sized external validation set is required to further confirm our research conclusions. Second, since lipidomics is a branch of systems biology, circulating lipoprotein subfractions mainly reflect an overall metabolic shift in cancer patients and may not reflect the metabolic states of the tumor cells alone. Therefore, in future studies, we plan to determine the relationship between abnormal lipoprotein metabolism and HCC development at the cellular level.

## 4. Materials and Methods

### 4.1. Ethical Statement

Prior to commencing the study, ethical approval was sought from the Research Ethics Committee of Tianjin Medical University Cancer Institute and Hospital in accordance with the 1964 Helsinki Declaration ethical standards (NO. bc2020098). Written informed consent was obtained from all participants, and the study was approved by the local Ethical Board.

### 4.2. Patients and Sample Collection

A total of 197 serum samples were enrolled at Tianjin Medical University Cancer Institute and Hospital (Tianjin, China) from July 2018 to December 2020. All serum samples were collected from 7:00 to 8:00 in the morning after the participants had fasted for at least 6 h. The samples were collected from all the patients who were initially diagnosed without liver disease-related treatment. The Barcelona Clinic Liver Cancer (BCLC) staging system was used to assess tumor stage. In the training set, we collected 51 patients with early-stage HBV-related HCC (BCLC stage 0-A) before surgical treatment, 37 patients with HBV-related hepatitis and HBV-related cirrhosis (hereafter referred to as liver disease, LD), and 50 NCs (with normal liver biochemistry, no type of malignancy or history of other benign disease, alcohol abuse and viral hepatitis). To identify the lipoprotein profile and establish a diagnostic model of HCC, a validation set was built from independent early-stage HCC (*n* = 24) and LD (*n* = 17) patient serum samples collected in the same way as those used in the training set. In addition, we selected serum AFP-negative patients (AFP level < 20 ng/mL) in the HCC and LD groups and collected 18 HCC patients with negative serum AFP expression for the next analysis. Next, eight pairs of cancerous and paracancerous tissue samples of HBV-related HCC patients from the validation set were obtained from surgical resections at Tianjin Medical University Cancer Institute and Hospital from May 2020 to August 2020. The inclusion and exclusion criteria, sample collection and storage are shown in the Supplementary Materials and methods. The collected test tube containing the blood sample was placed in a centrifuge at 4 °C and centrifuged at 3000 rpm for 15 min. Then, 400 μL of serum was collected from the upper layer of the test tube and stored at −80 °C until required for NMR detection. Tissue samples were fixed in 10% formalin and embedded in paraffin. The paraffin-sectioned tissues were serially cut into 5 μm sections and preserved at room temperature until required for mass spectrometric measurement.

### 4.3. Inclusion Criteria and Exclusion Criteria

The diagnoses of HCC, hepatitis and cirrhosis were based on the American Association for the Study of Liver Diseases (AASLD) Practice Guidelines.

The inclusion criteria were as follows:

1. Primary HCC diagnosed by histological or cellular examination.
2. Single tumor (regardless of size) or the number of tumors is less than 3 and the maximum diameter is ≤ 3 cm, and no history of portal invasion or extrahepatic spread.
3. HCC, cirrhosis and hepatitis with a history of HBV infection confirmed by virological assay.
4. Age > 18 years.
5. No previous treatment for HCC.
6. Knowledge of the study and agreement to follow-up.

Participants were excluded from the study if they met any of the following conditions:

1. History of other diagnosed malignancies.
2. History of anticancer treatment for HCC.
3. History of hepatitis virus infection without HBV.
4. Factors can cause abnormal elevation of serum AFP in normal controls, including pregnancy and any type of liver disease.
5. Participants with severe illnesses, including cardiovascular disease, endocrine disease and renal impairment.
6. Participants with lactation, current smoking and drug dependence.
7. Participants were taking lipid-lowering, hyperglycemic, anti-inflammatory, antithrombotic medications, dietary supplements, or antihypertensive treatment.

### 4.4. Magnetic Resonance Experiments

A Bruker 600 MHz NMR spectrometer was applied to estimate the lipoprotein subfractions. The Bruker IVDr lipoprotein subclass analysis (B.I.-LISA) method was used to predict the subfractions of lipoproteins for the analysis. Bruker’s Quant Ref manager within Top Spin was used to normalize the spectra to the same quantitative scale, and the spectral intensity was normalized to the proton concentration in units of millimoles per liter. First, Topspin 3.6.0 was used to calibrate the chemical shift to the methyl signal of trimethylsilyl propanoic acid (TSP), and then the alanine doublet was calibrated to 1.48 ppm; this method requires integration of the lipoprotein -CH3 and CH2- signals appearing in the 1D ^1^H NMR spectrum with chemical shifts of 0.8 and 1.25 ppm, respectively (Figure S1). The ^1^H-NMR platform has good intralaboratory repeatability and interlaboratory repeatability [11], and all tests were blind to the disease status of participants.

Lipoprotein subfractions were determined based on one-dimensional nuclear Overhauser effect spectroscopy (NOESY) magnetic resonance (MR) spectra using a partial least-squares regression model. Each lipoprotein class was further subdivided into subfractions according to its density: very-low-density lipoprotein (VLDL) was divided into VLDL 1–5, low-density lipoprotein (LDL) into LDL 1–6 and high-density lipoprotein (HDL) into HDL 1–4, with larger numbers indicating increasing density. Serum lipoprotein particle numbers (PNs) and serum concentrations of TG, cholesterol (CH), free cholesterol (FC), phospholipids (PL), apolipoprotein A1 (Apo-A1), apolipoprotein A2 (Apo-A2), and apolipoprotein B (Apo-B), as well as in each of the lipoprotein classes of VLDL, LDL, intermediate-density lipoprotein (IDL) and HDL, were estimated using a regression model developed by Bruker BioSpin. Finally, a dataset constructed from 112 variables was used in this study. Four-letter abbreviations were used to represent the variables; for example, the estimated VLDL-1 content of phospholipids was named V1PL, and the estimated total serum cholesterol was named TPCH. The NMR lipoproteins and subfractions are shown in Table S1.

### 4.5. Nanoscale Liquid Chromatography-Tandem Mass Spectrometry (Nano-LC-MS/MS) Analysis

Orbitrap Q-Exactive HF mass spectrometry (Thermo Fisher Scientific, Waltham, MA, USA) accompanied by a Thermo Scientific UltiMate 3000 UHPLC system was used to acquire lysed peptide sample data. Peptides were redissolved in loading buffer (2% ACN) with iRT standards (Biognosys, Schlieren, Switzerland) and separated using a 150-min gradient method (0–3 min, 3 to 9% buffer B; 3–127 min, 9 to 63% buffer B; 127–131 min, 63% buffer B; 131–149 min, 63 to 3% buffer B). The digested peptides were ionized at 2 kV, and mass spectrometry analysis data were collected using data-independent acquisition (DIA) mode. Full-scan MS1 acquisition was performed by an Orbitrap mass analyzer (scan range 300–1400 *m*/*z*) at a high resolution of 120,000. For MS2 acquisition, the spectra were recorded in top speed mode with a duty cycle time of 3 s. Precursor ions were selected and fragmented using higher-energy collisional dissociation (HCD) with 32% normalized collision energy. The maximum ion injection time for the MS2 scan was set to 35 ms, and the dynamic exclusion for the selected ions was 60 s. All tests were blind to the disease status of participants.

### 4.6. Statistical Analysis

#### 4.6.1. Multivariable and Univariable Statistical Analysis of NMR Data

Due to the hypothesized biological mechanisms between lipid fractions and HCC development, multivariable data analysis based on the projection principle was applied for statistical analysis of the ^1^H-NMR dataset. A pattern-recognition method that can discriminate between groups even in the presence of highly structured noise or confounding factors, unsupervised principal component analysis (PCA) and supervised partial least squares-discriminant analysis (PLS-DA), were implemented to analyze the raw data and classify the samples. Then, orthogonal partial least-squares discriminant analysis (OPLS-DA) was used to extract the correlated variables and optimize the maximum separation by using the Simca version 14.1 software package (UmetricsAB, Umea, Sweden).

The models were validated using 7-fold cross-validation to quantitatively assess their generalization ability and acquire robust statistical models. In 7-fold cross-validation, the dataset is split into seven equal-sized subsets. In each round, one subset is used for validation, and the remaining six subsets are used for training; this process is repeated seven times. The goodness-of-fit parameters and R^2^ and Q^2^ values calculated with 7-fold cross-validation as well as with cross-validated analysis of variance (CV-ANOVA, where *p* < 0.05 suggests the model is superior to one chosen at random) were obtained to measure the robustness and quality of the models. The associated R^2^ and Q^2^ parameters represent the interpretation rate of the matrix and model predictive capability; the closer the metrics are to 1, the larger the variance explained by the model and the more reliable its predictive power. Furthermore, a permutation test (999 permutations) was performed to validate the degree of overfitting based on the values of the R^2^-intercept and Q^2^-intercept. The reproducibility and robustness of each model were validated by the Q^2^-intercept; the more negative the value of the Q^2^-intercept was, the better the performance of the model.

In the OPLS-DA model, most of the variables related to the classification were concentrated in the direction of the first predicted principal component. To identify the differential lipoprotein subfractions, the VIP scores calculated by the OPLS-DA model were used to reflect the most influential contribution of each variable to the model. When VIP > 1.0, the variable was considered potentially relevant. Differences in lipoprotein subfractions between the three groups were assessed by the Kruskal–Wallis test (nonnormally distributed data) in the training sets, and *p* < 0.05 was considered statistically significant. Lipoprotein subfractions with VIP scores > 1.0 and *p* < 0.05 were selected and entered into a binary logistic regression model to design the best lipoprotein subfraction combination. To further evaluate the diagnostic performance of the potential biomarkers, receiver operating characteristic (ROC) curves were analyzed to evaluate the accuracy of this model. Each biomarker panel’s diagnostic performance was evaluated by using the area under the ROC curve (AUC) and the sensitivity and specificity at the optimal cutoff point defined by the minimum distance to the top-left corner of the ROC curve graph. For the participants’ clinical characteristics, the Mann–Whitney U test was used to compare continuous variables, and Pearson’s chi-square test was used to compare categorical variables. Correlations were calculated by Spearman rank correlation analysis, and *p* < 0.05 was considered statistically significant. Logistic regression and statistical analysis were performed by using IBM SPSS version 26.0 (SPSS Inc., Armonk, NY, USA).

#### 4.6.2. Quantification and Statistical Analysis of LC-MS/MS Data

The DIA data were searched using the Human-specific UniProt database (20,365 sequences), and LC-MS/MS data were analyzed by Spectronaut (v14.5.200813.47784). The library was generated using the default settings for trypsin/P digestion rules and high protein and peptide confidential levels [false discovery rate (FDR) = 0.01]. The output-quantified protein intensities were processed using Spectronaut, and a median normalization procedure was applied to normalize the data. Proteins with at least 30% appearance in all samples were chosen for the subsequent analysis, and missing values were replaced with half of the minimum value of each protein intensity. A fold-change of > 1.5 or <0.5 and a *p*-value < 0.05 (The Mann–Whitney U test) were set as cutoff values for the differential proteins. The protein corresponding gene and OS information of 371 cancer samples from TCGA were applied to generate survival curves with the survival and survminer packages in the R package (version 3.6.0). Gene Ontology (GO) functional annotations and Kyoto Encyclopedia of Genes and Genomes (KEGG) pathway enrichment were performed using the R package (clusterProfiler, v3.16.1) and the org.Hs.eg.db (v3.11.4) annotation database. The background genes were set to all quantified genes, and the differential genes were input to generate the enrichment pathway list and figures.

## 5. Conclusions

In conclusion, this study aimed to objectively assess the clinical applicative value of serum lipoprotein subfraction testing in the diagnosis of HBV-related HCC patients with BCLC stage 0-A. The results clearly indicate that the lipidomic biomarker panel constructed with the particle numbers of VLDL, IDL, LDL-1, LDL-2, LDL-3, LDL-4, LDL-5, and LDL-6 could be used in the diagnosis of HCC. Meanwhile, we found that LPL transcript levels in cancerous tissues of HCC patients showed a significant increase compared with paracancerous tissues, and the high expression of LPL showed a significant positive correlation with the poor prognosis of patients by bioinformatic analysis. Moreover, LC-MS/MS analysis indicated that abnormal lipid metabolism is an important influential factor in potentially promoting HBV-related HCC development. Our study focuses on an innovative combination of alterations in the lipid profile of cancer patients and ^1^H-NMR-based lipidomics research, which provides new insight for the development of diagnostic and prognostic biomarkers for HBV-related HCC with BCLC stage 0-A.

However, several limitations to this pilot study need to be considered. For example, this study lacks a large number of external verification samples to further verify the generalizability of the diagnostic panel. Despite the relatively limited sample size, the study certainly adds to our understanding of the use of serum lipoprotein biomarkers in the diagnosis of HCC. Further large prospective studies with external validation should be undertaken to determine whether this lipidomic panel may improve surveillance and management strategies in patients with HCC.

## Figures and Tables

**Table 1:** Clinical characteristics of the HCC group and LD group.

	Training Set			Validation Set		
Characteristics	HCC	Liver Disease	*p*-Value	HCC	Liver Disease	*p*-Value
*n*	51	37		24	17	
Age (years)	58 (33.00 to 71.00)	59 (43.00 to 66.00)	0.889	60.50 (51.75 to 66.00)	49.00 (45.50 to 58.00)	0.010
Sex (male/female)	37/14	21/16	0.123	16/8	8/9	0.209
AFP (ng/mL)	23.88 (4.80 to 126.20)	2.69 (1.68 to 4.64)	<0.001	60.50 (3.42 to 481.00)	2.66 (1.98 to 3.55)	<0.001
ALT (IU/L)	36.00 (22.00 to 48.00)	25.00 (16.00 to 36.50)	0.018	24.00 (15.25 to 31.75)	21.00 (14.00 to 35.00)	0.937
AST (IU/L)	37.00 (26.00 to 59.00)	28.00 (19.00 to 36.50)	0.002	29.00 (21.50 to 38.50)	21.00 (16.00 to 29.00)	0.095
ALB (g/L)	41.60 (39.40 to 45.50)	43.50 (37.50 to 46.95)	0.422	42.55 (36.50 to 45.08)	44.90 (41.45 to 47.60)	0.058
TP (g/L)	74.50 (70.90 to 77.20)	68.40 (53.50 to 77.65)	0.016	70.45 (64.60 to 75.93)	74.40 (66.25 to 76.25)	0.404
TBIL (μmmol/L)	16.50 (13.90 to 23.60)	14.50 (11.05 to 18.63)	0.154	14.70 (11.08 to 20.98)	12.40 (9.80 to 18.25)	0.375
CRE (μmol/L)	61.00 (53.00 to 69.00)	62.00 (52.00 to 79.50)	0.244	61.50 (56.00 to 78.00)	60.00 (51.50 to 68.00)	0.255
BCLC stage						
stage 0	9	/		1	/	
stage A	42	/		23	/	
Child-Pugh class						
A	46	33		22	16	
B-C	5	4		2	1	
Tumor diameter (cm)						
≤3	25	/		10	/	
>3	26	/		14	/	

*p*-values: Mann–Whitney U test for continuous variables and Pearson’s chi-square test for categorical variables. Continuous data are presented as medians with interquartile ranges (IQRs). Abbreviations: AFP, alpha-fetoprotein; ALT, alanine aminotransferase; AST, aspartate transaminase; ALB, albumin; TP, total protein; TBIL, total bilirubin; CRE, creatinine.

**Table 2:** Summary of the lipoprotein subfraction statistical data from OPLS-DA analysis from HCC patients, liver disease patients, and normal controls.

			HCC vs. LD		HCC vs. NCs		
Index	Description	Unit	VIP	*p* (corr)	VIP	*p* (corr)	*p*-Value
TPCH	Total Cholesterol	mg/dL	1.045	−0.026	1.030	0.165	0.596
HDCH	HDL-C	mg/dL	0.414	0.120	1.032	0.703	2.931 × 10^−14^
TPA1	Apo-A1	mg/dL	1.089	0.202	1.146	0.631	3.742 × 10^−10^
TBPN	Total Particle Number	nmol/L	3.451	−0.006	3.512	0.107	0.775
VLPN	VLDL Particle Number	nmol/L	1.083	0.014	1.329	0.310	0.003
IDPN	IDL Particle Number	nmol/L	2.514	−0.347	1.379	0.277	6.318 × 10^−5^
LDPN	LDL Particle Number	nmol/L	3.465	0.069	2.772	−0.016	0.339
L1PN	LDL-1 Particle Number	nmol/L	3.743	−0.310	3.024	−0.512	1.399 × 10^−9^
L2PN	LDL-2 Particle Number	nmol/L	2.612	−0.209	2.118	−0.511	1.712 × 10^−4^
L3PN	LDL-3 Particle Number	nmol/L	1.451	−0.032	3.144	−0.838	<0.001
L4PN	LDL-4 Particle Number	nmol/L	2.708	0.221	1.645	−0.443	2.148 × 10^−10^
L5PN	LDL-5 Particle Number	nmol/L	3.232	0.296	2.737	0.643	5.121 × 10^−8^
L6PN	LDL-6 Particle Number	nmol/L	2.971	0.218	5.050	0.934	<0.001
HDA1	HDL Apo-A1	mg/dL	1.037	0.167	1.227	0.644	1.538 × 10^−11^
L1CH	LDL-1 Cholesterol	mg/dL	1.201	−0.316	0.993	−0.563	2.456 × 10^−9^
L6CH	LDL-6 Cholesterol	mg/dL	0.942	0.227	1.324	0.936	<0.001
L6AB	LDL-6 Apo-B	mg/dL	0.802	0.218	1.184	0.934	<0.001
H1A1	HDL-1 Apo-A1	mg/dL	1.229	0.230	0.146	−0.271	0.011
H4A1	HDL-4 Apo-A1	mg/dL	0.811	−0.083	1.306	0.773	<0.001

The characteristics of significantly different variables in the OPLS-DA model. *p* (corr) is the OPLS-DA loading scaled as a correlation coefficient. The significance of the values was assessed using the Kruskal–Wallis test. VIP, variable importance in projection; HCC, hepatocellular carcinoma; LD, liver disease; NCs, normal controls.

**Table 3:** Summary of the lipoprotein subfraction statistical data from OPLS-DA analysis from AFP (-) HCC patients, AFP (-) liver disease (LD) patients, and normal controls (NCs).

			AFP (-) HCC vs. AFP (-) LD		AFP (-) HCC vs. NCs		
Index	Description	Unit	VIP	*p* (corr)	VIP	*p* (corr)	*p*-Value
TPCH	Total Cholesterol	mg/dL	1.160	0.105	0.836	0.281	0.463
TPTG	Total Triglycerides	mg/dL	1.456	−0.450	1.047	0.060	0.075
LDCH	LDL-C	mg/dL	1.274	0.373	0.204	−0.013	0.214
HDCH	HDL-C	mg/dL	0.414	0.261	1.207	0.763	<0.001
TPA1	Apo-A1	mg/dL	0.690	0.294	1.307	0.655	5.091 × 10^−14^
TBPN	Total Particle Number	nmol/L	3.986	0.138	2.477	0.163	0.594
VLPN	VLDL Particle Number	nmol/L	1.794	−0.490	1.850	0.271	0.005
IDPN	IDL Particle Number	nmol/L	1.919	−0.537	2.066	0.309	1.076 × 10^−5^
LDPN	LDL Particle Number	nmol/L	4.282	0.350	0.463	0.035	0.084
L1PN	LDL-1 Particle Number	nmol/L	3.702	−0.747	3.206	−0.422	9.240 × 10^−8^
L2PN	LDL-2 Particle Number	nmol/L	1.467	−0.354	1.715	−0.465	1.573 × 10^−4^
L3PN	LDL-3 Particle Number	nmol/L	1.137	−0.110	3.224	−0.849	<0.001
L4PN	LDL-4 Particle Number	nmol/L	2.181	0.382	2.018	−0.511	1.325 × 10^−11^
L5PN	LDL-5 Particle Number	nmol/L	3.338	0.746	2.940	0.610	7.372 × 10^−9^
L6PN	LDL-6 Particle Number	nmol/L	3.419	0.685	5.325	0.939	<0.001
HDA1	HDL Apo-A1	mg/dL	0.703	0.298	1.418	0.675	4.663 × 10^−14^
LDAB	LDL Apo-B	mg/dL	1.004	0.350	0.109	0.035	0.084
L1CH	LDL-1 Cholesterol	mg/dL	1.149	−0.738	1.013	−0.475	1.382 × 10^−8^
L6CH	LDL-6 Cholesterol	mg/dL	0.942	0.717	1.398	0.930	<0.001
L6PL	LDL-6 Phospholipids	mg/dL	0.709	0.757	1.006	0.932	<0.001
L6AB	LDL-6 Apo-B	mg/dL	0.802	0.685	1.249	0.939	<0.001
H4A1	HDL-4 Apo-A1	mg/dL	0.811	0.421	1.335	0.760	<0.001

The characteristics of significantly different variables in the OPLS-DA model. *p* (corr) is the OPLS-DA loading scaled as a correlation coefficient. The significance of the values was assessed using the Kruskal–Wallis test. VIP, variable importance in projection; AFP (-) HCC, AFP-negative hepatocellular carcinoma; AFP (-) LD, AFP-negative liver disease; NCs, normal controls.

**Table 4:** Changes in relative levels of lipoprotein subfractions in serum samples from HCC patients, liver disease patients and normal controls.

	HCC vs. LD		HCC vs. NCs			AFP (-) HCC vs. AFP (-) LD		AFP (-) HCC vs. NCs		
Index	VIP	Trend	VIP	Trend	*p*-Value	VIP	Trend	VIP	Trend	*p*-Value
VLPN	1.083	up	1.329	down	3.023 × 10^−3^	1.794	up	1.850	down	4.733 × 10^−3^
IDPN	2.514	up	1.379	down	6.318 × 10^−5^	1.919	up	2.066	down	1.076 × 10^−5^
L1PN	3.743	up	3.024	up	1.398 × 10^−8^	3.702	up	3.206	up	9.240 × 10^−8^
L2PN	2.613	up	2.118	up	1.712 × 10^−4^	1.467	up	1.715	up	1.573 × 10^−4^
L3PN	1.451	down	3.144	up	<0.001	1.137	down	3.224	up	<0.001
L4PN	2.708	down	1.645	up	2.148 × 10^−10^	2.181	down	2.018	up	1.325 × 10^−11^
L5PN	3.232	down	2.737	down	5.121 × 10^−8^	3.338	down	2.940	down	7.372 × 10^−9^
L6PN	2.971	down	5.050	down	<0.001	3.419	down	5.325	down	<0.001

The common variables between the two constructed OPLS-DA models were selected if their VIP scores > 1.0 and univariable *p*-values < 0.05. HCC, hepatocellular carcinoma; NCs, normal controls; VIP, variable importance in projection.

**Table 5:** Test performance characteristics for the signature panel.

Experiment Set	Group	Dataset	AUC (95% CI)	Sensitivity (%)	Specificity (%)
Training set	HCC vs. LD	AFP	0.831 (0.736 to 0.902)	74.51	81.08
		panel	0.850 (0.758 to 0.917)	88.24	72.97
		Panel + AFP	0.861 (0.771 to 0.926)	88.24	75.68
	HCC vs. NCs	panel	1.000 (0.964 to 1.000)	100.00	100.00
Validation set	HCC vs. LD	AFP	0.833 (0.684 to 0.931)	66.67	100.00
		panel	0.926 (0.800 to 0.984)	83.33	100.00
		Panel + AFP	1.000 (0.914 to 1.000)	100.00	100.00
AFP-negative	HCC vs. LD	panel	0.773 (0.680 to 0.850)	69.23	76.00
	HCC vs. NCs	panel	1.000 (0.964 to 1.000)	100.00	100.00

AUC, area under the receiver operating curve; CI, confidence interval; HCC, hepatocellular carcinoma; LD, liver disease; NCs, normal controls.

## Supplementary Materials

The following are available online at https://www.mdpi.com/article/10.3390/jpm11111143/s1. Figure S1: Stacked view of the ^1^H-NMR spectra of lipoprotein subfractions. HCC patient (blue), benign liver disease patient (green) and normal control (red). Figure S2: The PCA and PLS-DA score plot in the training set. HCC patient (red), benign liver disease patient (green) and normal control (yellow). Figure S3: PCA and PLS-DA score plot of serum AFP-negative patients. HCC patient (red), benign liver disease patient (green) and normal control (yellow). Figure S4: The correlation of serum AFP and selected lipoprotein particles was analyzed using Spearman rank correlation analysis. Figure S5: The correlation of selected lipoprotein particles and clinical features was analyzed using Spearman rank correlation analysis. Figure S6: ROC curve analysis following binary logistic regression in distinguishing HCC patients from non-HCC patients. The diagnostic performance of the lipidomic biomarker panel in discriminating (a) HCC from non-HCC patients and (b) AFP (-) HCC from AFP (-) non-HCC patients. Figure S7: Comparison of DEPs associated with lipid metabolism between cancerous and paracancerous tissue samples. In eight patients, 11 differential proteins showed significant differences. The Mann–Whitney U test was performed to determine whether the differences were statistically significant. N: paracancerous HCC tissues, T: cancerous HCC tissues. Figure S8: Relative expression levels of 11 differentially expressed proteins between cancerous and paracancerous HCC tissues. The data are expressed as medians with interquartile ranges and were analyzed by the Mann–Whitney U test (* *p* < 0.05, ** *p* < 0.001, *** *p* < 0.0001). N: paracancerous HCC tissues, T: cancerous HCC tissues. Figure S9: Results of KEGG pathway enrichment analysis. The enriched pathways of upregulated (red) and downregulated (blue) DEPs associated with lipid metabolism. Figure S10: The prognostic value of DEPs for HCC patients. The overall survival curves (a) and disease-free survival curves (b) for HCC patients with high (red) and low expression levels of (blue) MBOAT7, GDP2, and ACADS were plotted using the GEPIA database (http://gepia2.cancer-pku.cn/ accessed on 23 August 2021) at the threshold *p*-value of < 0.05. Table S1: NMR lipoproteins & subfractions. Table S2: Clinical characteristics of the HCC group and normal control group. Table S3: Clinical characteristics of the AFP-negative HCC group and AFP-negative LD group. Table S4: List of calculated parameters in the training set from multivariable and univariable statistical analysis. Table S5: List of calculated parameters of AFP-negative patients from multivariable and univariable statistical analysis. Table S6: The absolute numbers of each lipoprotein subfraction. Table S7: Correlation between selected variables and clinical features of HCC patients. Table S8: Test performance characteristics for the signature panel. Table S9: Differentially expressed proteins between cancerous tissues and paracancerous tissues.
