# Supplementary material for: The Application Value of Lipoprotein Particle Numbers in the Diagnosis of HBV-Related Hepatocellular Carcinoma with BCLC Stage 0-A
Source: J Pers Med. 2021 Nov 4;11(11):1143. doi: 10.3390/jpm11111143 (PMC8617679; doi:10.3390/jpm11111143)
Supplement: Supplementary file 1 [file jpm-11-01143-s001.zip › Figure S9.pdf]

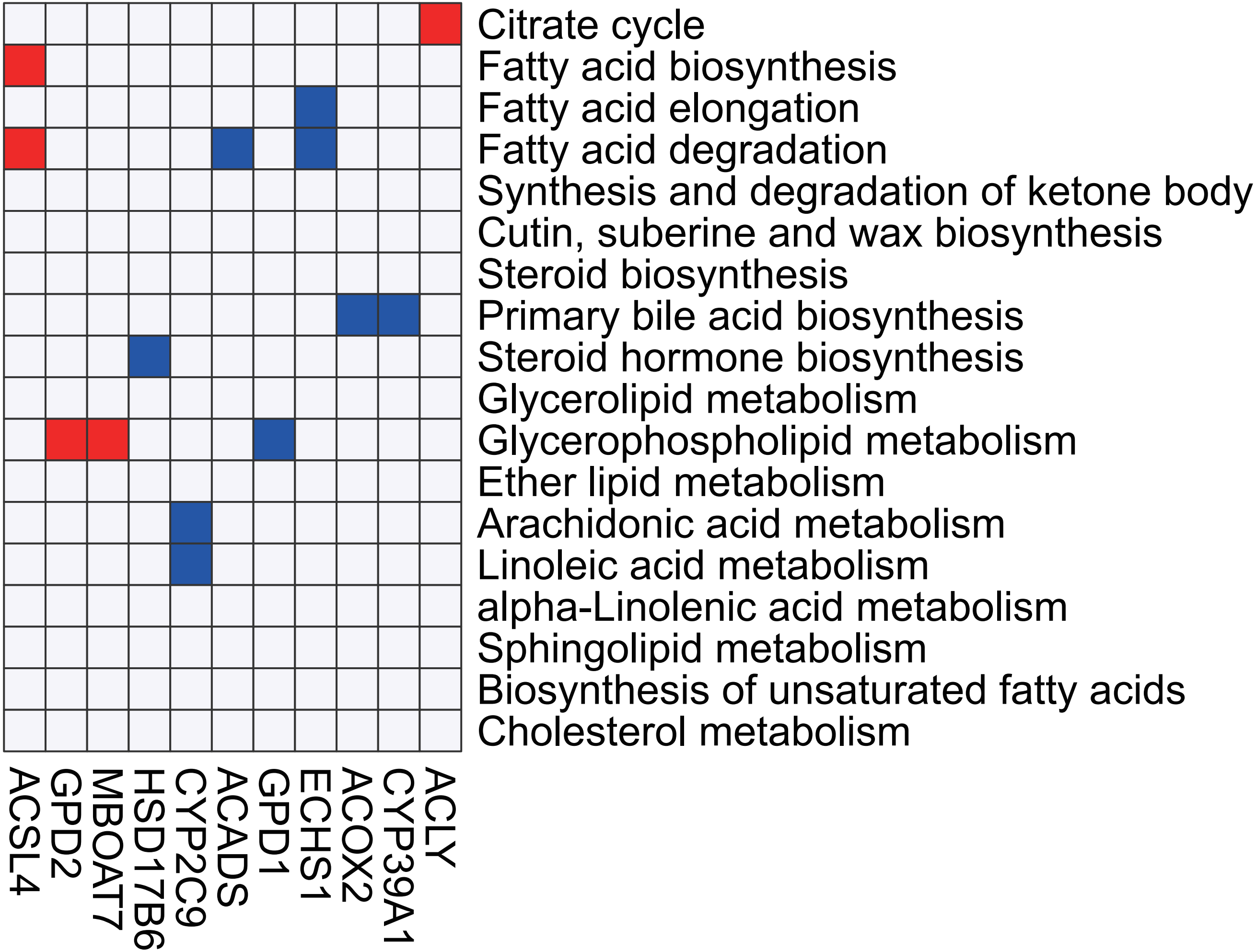
Citrate cycle
Fatty acid biosynthesis
Fatty acid elongation
Fatty acid degradation
Synthesis and degradation of ketone body
Cutin, suberine and wax biosynthesis
Steroid biosynthesis
Primary bile acid biosynthesis
Steroid hormone biosynthesis
Glycerolipid metabolism
Glycerophospholipid metabolism
Ether lipid metabolism
Arachidonic acid metabolism
Linoleic acid metabolism
alpha-Linolenic acid metabolism
Sphingolipid metabolism
Biosynthesis of unsaturated fatty acids
Cholesterol metabolism
ACSL4
GPD2
MBOAT7
HSD17B6
CYP2C9
ACADS
GPD1
ECHS1
ACOX2
CYP39A1
ACLY
