# Supplementary figures and images for: The Application Value of Lipoprotein Particle Numbers in the Diagnosis of HBV-Related Hepatocellular Carcinoma with BCLC Stage 0-A
Source: J Pers Med. 2021 Nov 4;11(11):1143. doi: 10.3390/jpm11111143 (PMC8617679; doi:10.3390/jpm11111143)

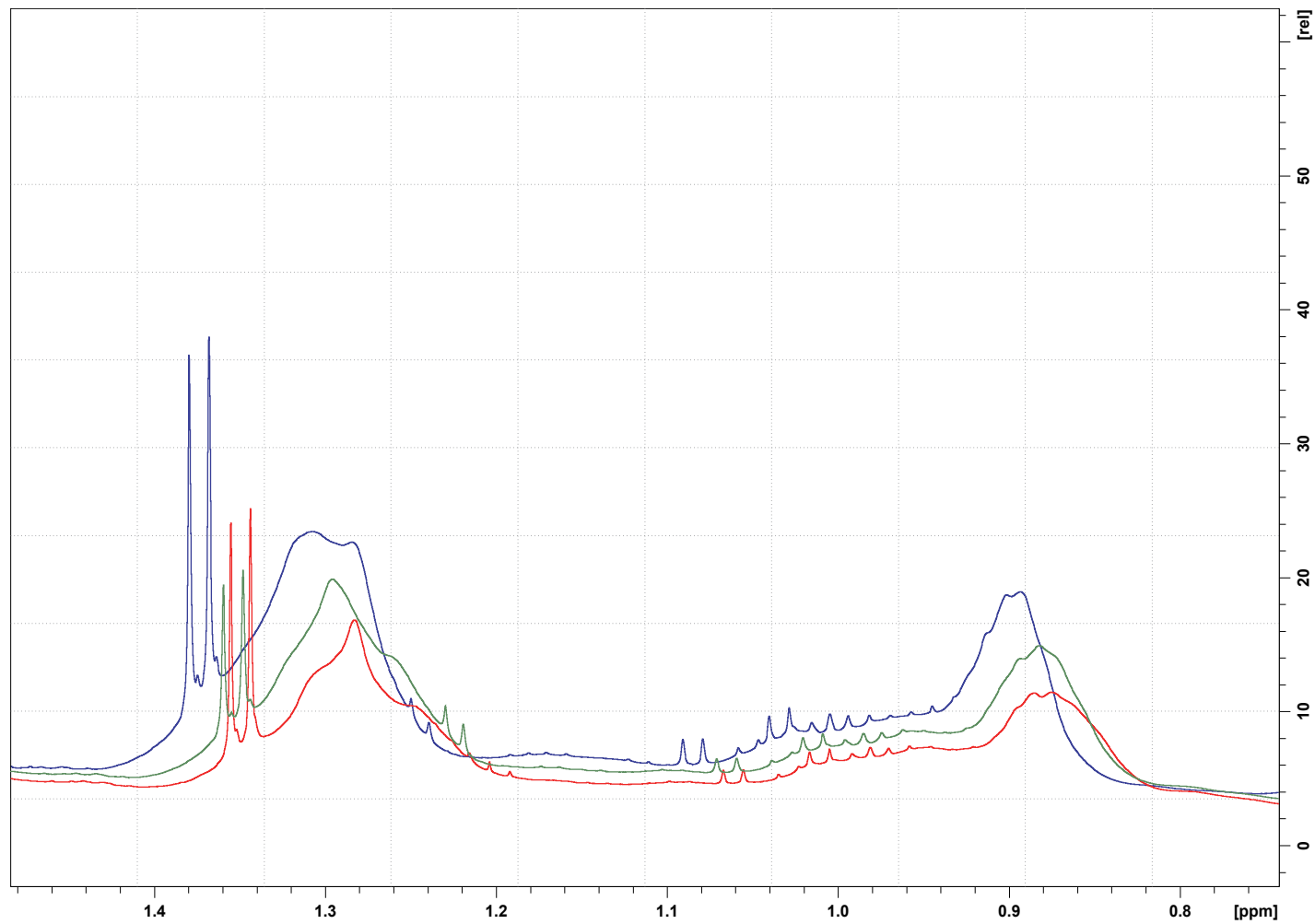

Supplement: Supplementary file 1 [file jpm-11-01143-s001.zip › Figure S1.pdf]

a

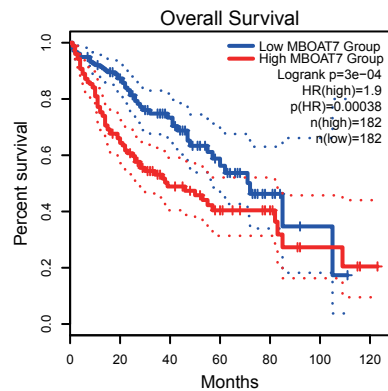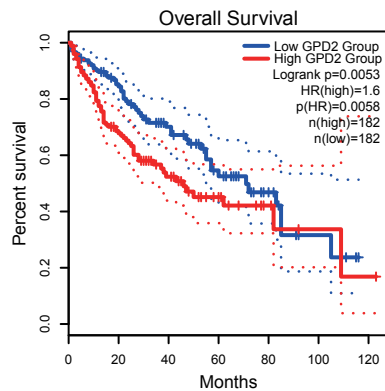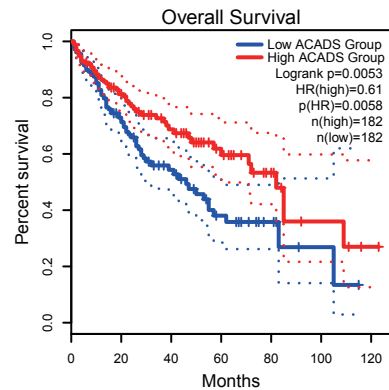

b

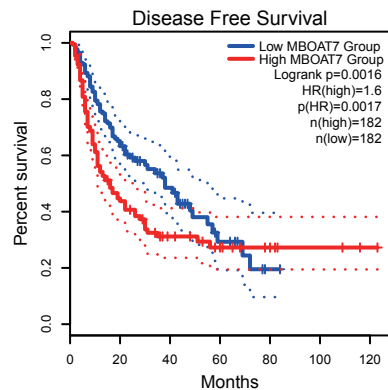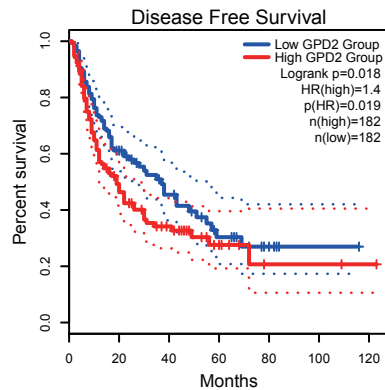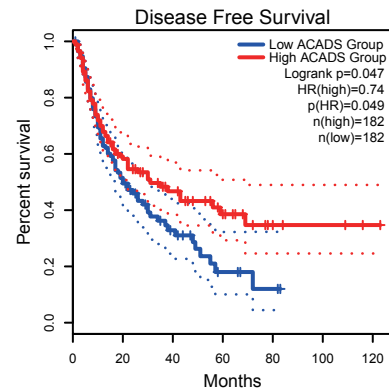

Supplement: Supplementary file 1 [file jpm-11-01143-s001.zip › Figure S10.pdf]

a

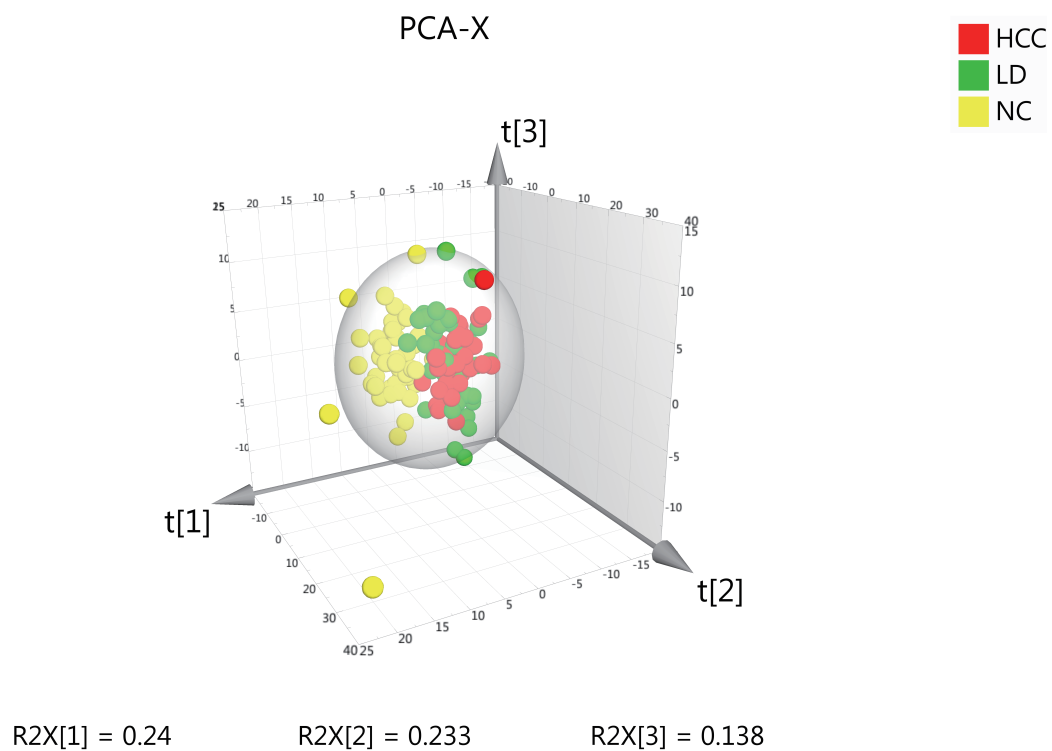

b

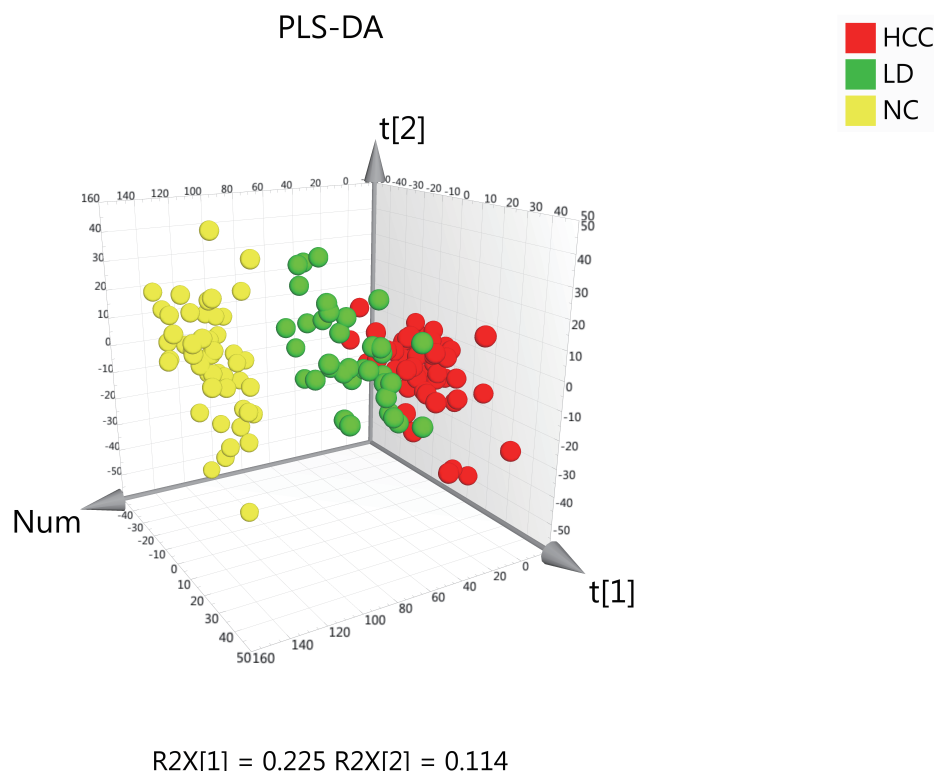

Supplement: Supplementary file 1 [file jpm-11-01143-s001.zip › Figure S2.pdf]

a

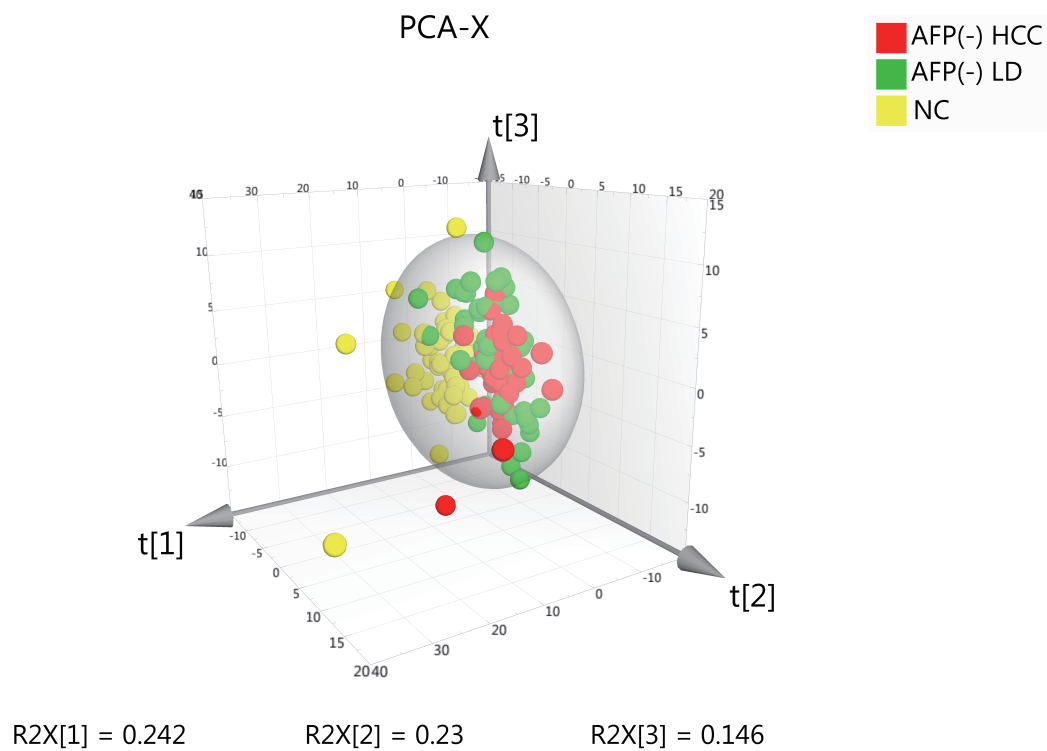

b

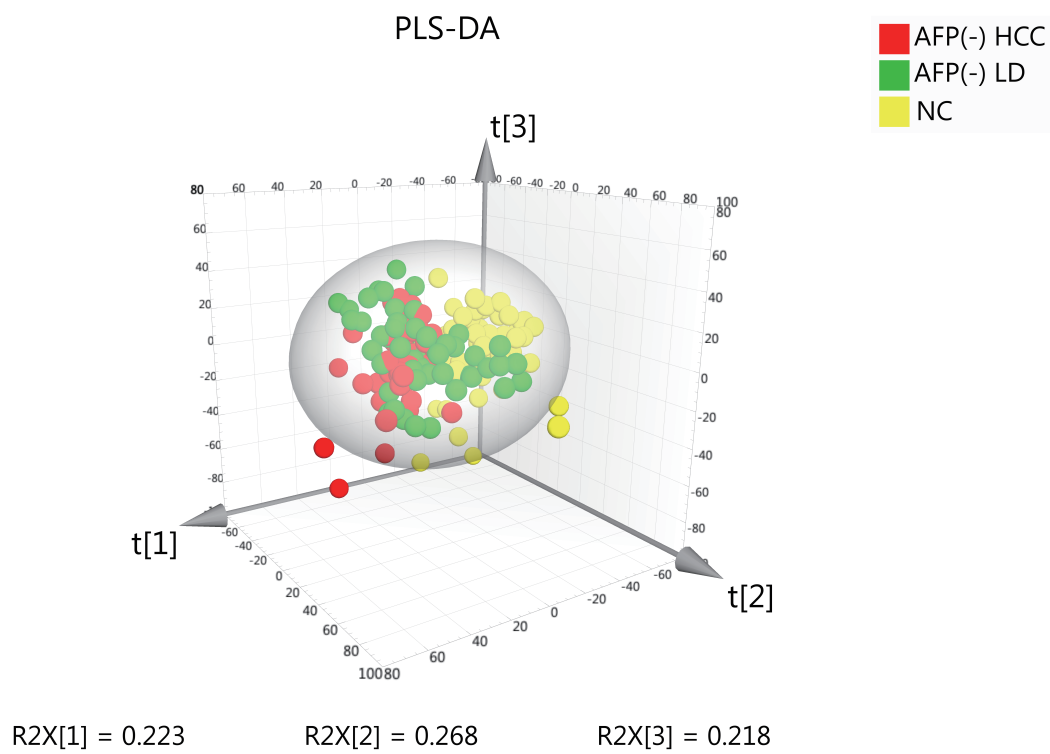

Supplement: Supplementary file 1 [file jpm-11-01143-s001.zip › Figure S3.pdf]

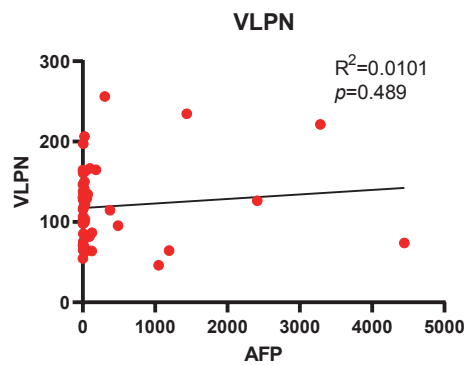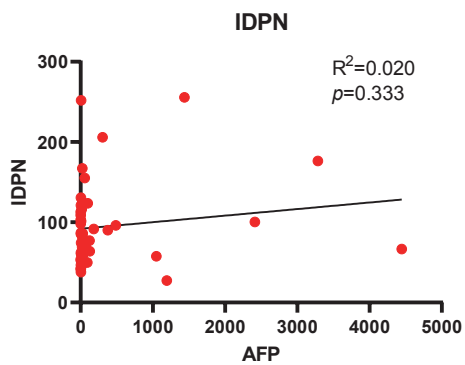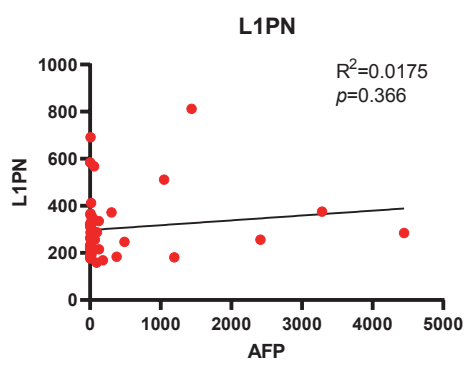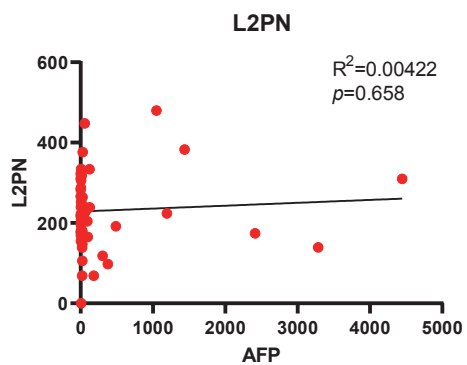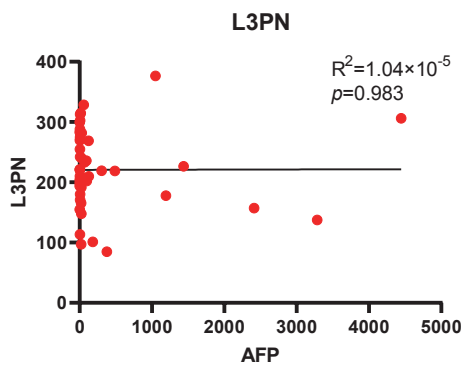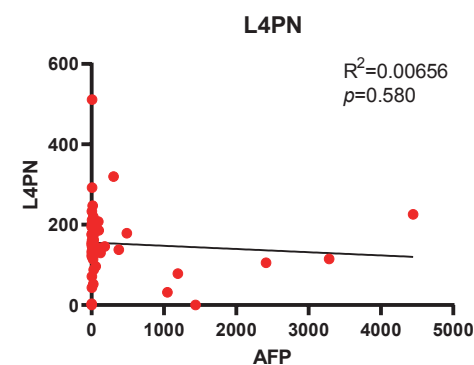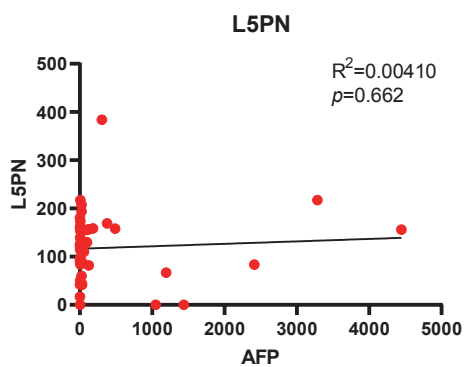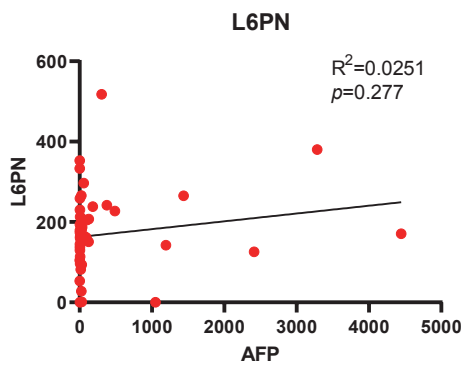

Supplement: Supplementary file 1 [file jpm-11-01143-s001.zip › Figure S4.pdf]

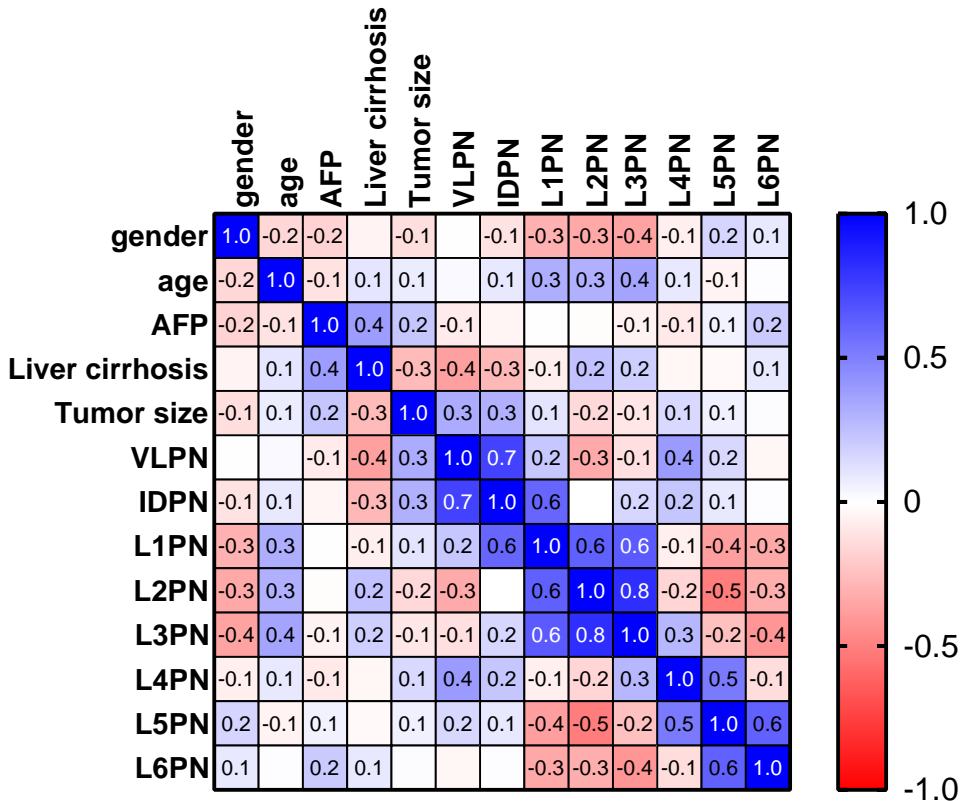

Supplement: Supplementary file 1 [file jpm-11-01143-s001.zip › Figure S5.pdf]

a

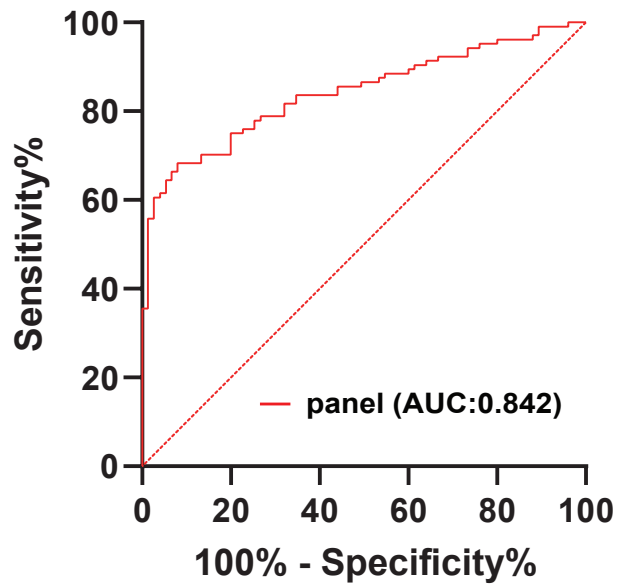

b

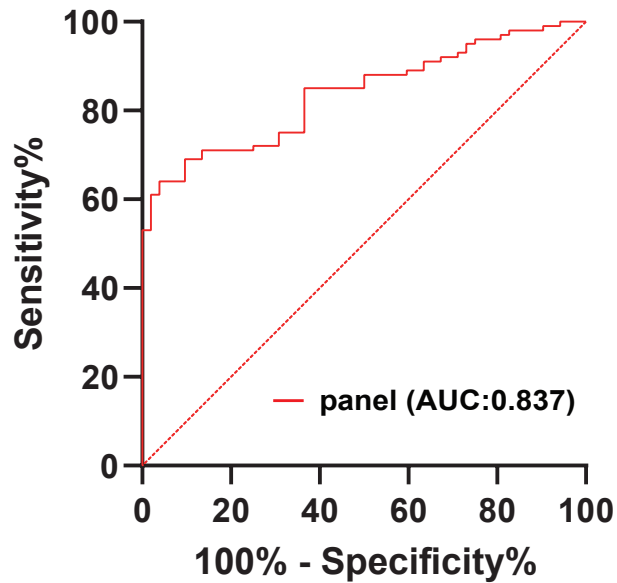

Supplement: Supplementary file 1 [file jpm-11-01143-s001.zip › Figure S6.pdf]

P=0.044

**ACSL4**

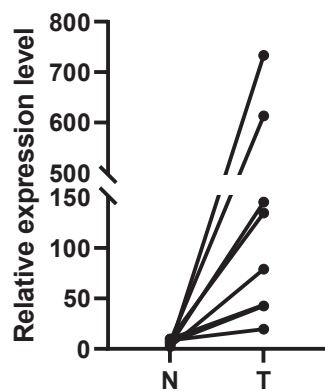

P=0.00087

**MBOA7**

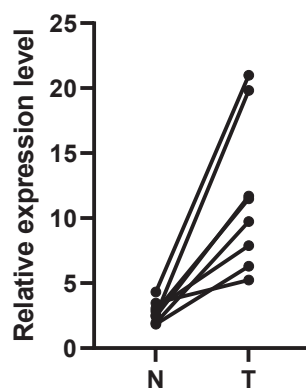

P=0.00015

**ACLY**

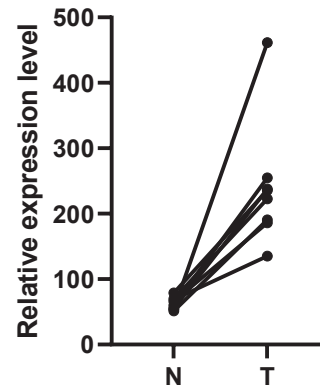

P<0.0001

**GPDM**

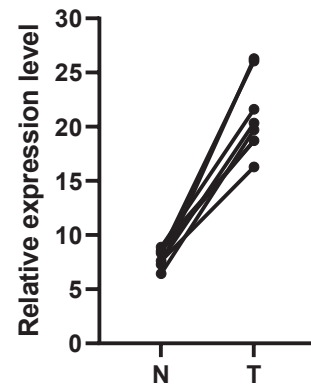

P=0.00027

**GPDA**

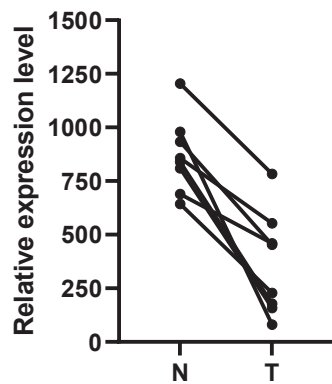

P<0.0001

**ACOX2**

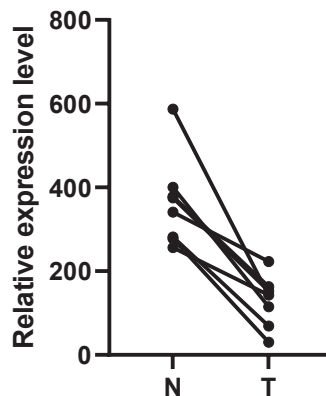

P<0.0001

**ECHM**

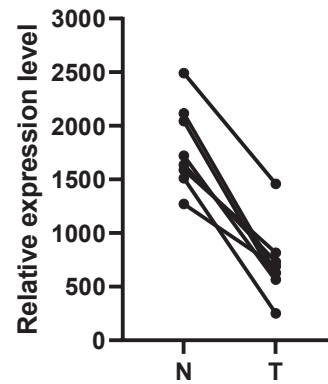

P=0.00011

**ACADS**

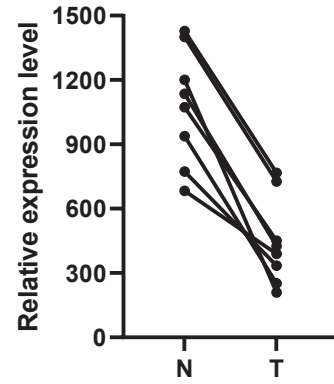

P<0.0001

**CP2C9**

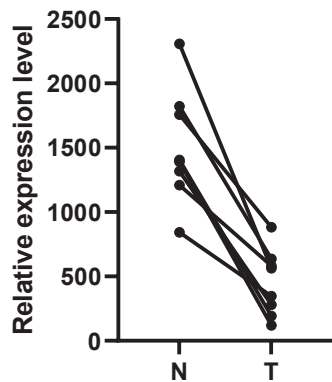

P=0.00015

**H17B6**

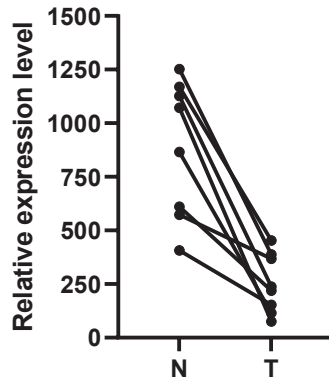

P=0.00018

**CP39A**

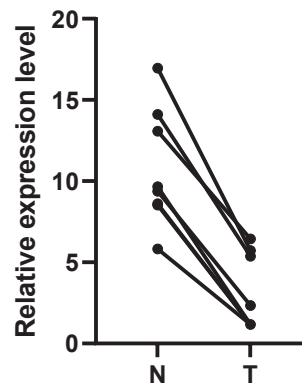

Supplement: Supplementary file 1 [file jpm-11-01143-s001.zip › Figure S7.pdf]

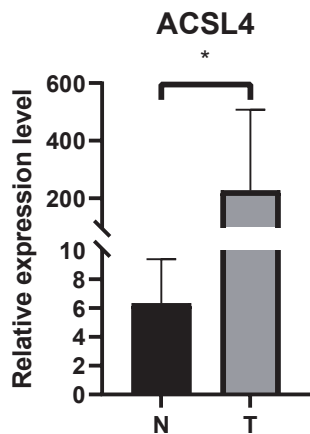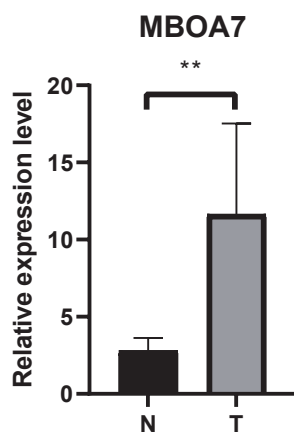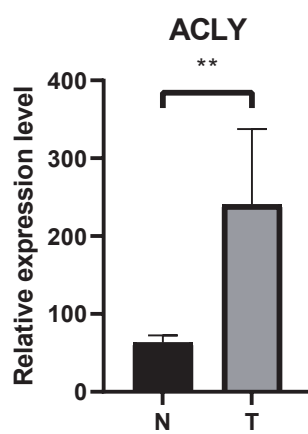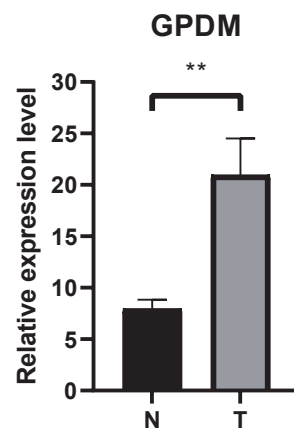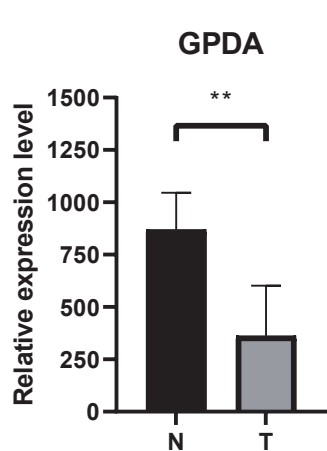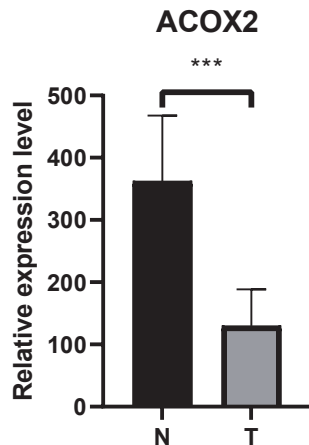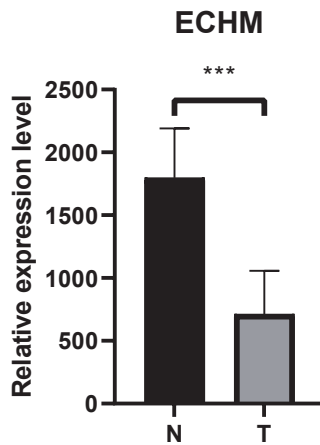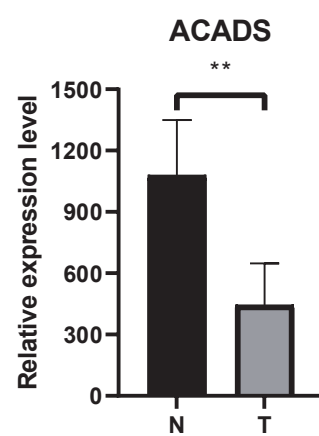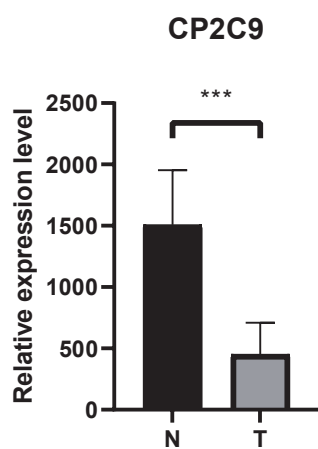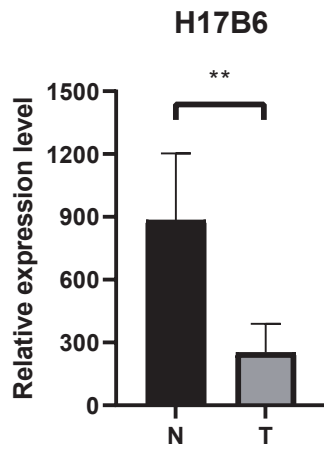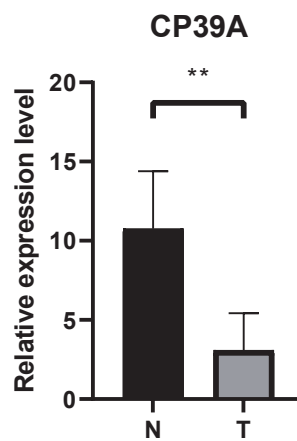

Supplement: Supplementary file 1 [file jpm-11-01143-s001.zip › Figure S8.pdf]
